# c-Src activity is differentially required by cancer cell motility modes

**DOI:** 10.1038/s41388-017-0071-5

**Published:** 2018-01-30

**Authors:** Jeremy S. Logue, Alexander X. Cartagena-Rivera, Richard S. Chadwick

**Affiliations:** 1 National Heart, Lung, and Blood Institute, National Institutes of Health, Bethesda, MD USA; 2 National Institute on Deafness and Other Communication Disorders, National Institutes of Health, Bethesda, MD USA; 3 Department of Regenerative and Cancer Cell Biology, Albany Medical College, Albany, NY USA

## Abstract

Cancer cell migration requires that cells respond and adapt to their surroundings. In the absence of extracellular matrix cues, cancer cells will undergo a mesenchymal to ameboid transition, whereas a highly confining space will trigger a switch to “leader bleb-based” migration. To identify oncogenic signaling pathways mediating these transitions, we undertook a targeted screen using clinically useful inhibitors. Elevated Src activity was found to change actin and focal adhesion dynamics, whereas inhibiting Src triggered focal adhesion disassembly and blebbing. On non-adherent substrates and in collagen matrices, amoeboid-like, blebbing cells having high Src activity formed protrusions of the plasma membrane. To evaluate the role of Src in confined cells, we use a novel approach that places cells under a slab of polydimethylsiloxane (PDMS), which is held at a defined height. Using this method, we find that leader bleb-based migration is resistant to Src inhibition. High Src activity was found to markedly change the architecture of cortical actomyosin, reduce cell mechanical properties, and the percentage of cells that undergo leader bleb-based migration. Thus, Src is a signal transducer that can potently influence transitions between migration modes with implications for the rational development of metastasis inhibitors.

## Introduction

During cancer metastasis, migrating cells encounter diverse environments. Because of this, metastasis requires that cells be inherently flexible. In environments rich in extracellular matrix (ECM) proteins, a member of the integrin family of proteins near the edge of an actin polymerization-driven plasma membrane protrusion will bind to a specific ECM protein (e.g., fibronectin and laminin). Once engaged with the ECM, activated integrins will recruit a host of proteins, including paxillin, focal adhesion kinase (FAK), Src, talin, vinculin, α-actinin, and many others to form a “focal adhesion” [1]. Within this complex, talin, vinculin, and α-actinin connect the focal adhesion to the actin cytoskeleton [2]. In fibroblasts, non-muscle myosin supplies the force to “tug” on integrins [3], which triggers focal adhesion maturation and pulls the cell forward. The tyrosine kinases FAK and Src form a signaling complex that begins with the auto-phosphorylation of FAK^Y397^. This site is recognized by the SH2 domain of Src, and FAK is subsequently phosphorylated on two additional sites, Y576 and Y577, leading to full activation of FAK. Fully activated FAK then goes on to phosphorylate other focal adhesion proteins, such as paxillin and p130Cas [4, 5]. In FAK null cells, focal adhesions are large because of a reduced turnover rate [6–9]. Because FAK and Src are important for determining focal adhesion dynamics, and, consequently, cancer cell proliferation, survival, and migration, they are the targets of some of the newer anticancer drugs (i.e., Dasatinib and Defactinib). However, for the treatment of solid tumors, these drugs and others have been met with limited success [10].

More recently, it was discovered that cancer cells are prone to an integrin-, or focal adhesion-, independent mode of migration. This mode of migration instead uses intracellular pressure-driven plasma membrane blebs. In contrast to the formation of specific integrin contacts on the ECM, this type of migration uses nonspecific friction with the extracellular environment [11]. In cancer cells, the intracellular pressure is increased relative to normal cells by high myosin contractility [12]. By inducing the phosphorylation of myosin light chain kinase, certain oncogenes, including BRAF V600E, are closely linked to the activation of myosin [13]. When intracellular pressure is sufficiently increased [14], a portion of the plasma membrane will spontaneously separate from the underlying actin cortex, thus forming a “bleb”. Under normal tissue culture conditions, blebs are quickly retracted, but, within the confines of tissues, cancer cells will form a very large persistent bleb, which has been shown to be required for migration in vivo and in vitro [11, 12, 15, 16]. Because these very large blebs lead the way during cancer cell migration, we refer to them as “leader blebs” [15]. Central to this type of migration is the continued flow of parallel actin fibers from the tip to the narrow constriction connecting the leader bleb to the rest of the cell, or cell body, which is driven by myosin [11, 12, 15, 16]. Importantly, when integrin binding or matrix metalloproteases (MMPs) are inhibited, cancer cells will undergo a “mesenchymal-to-amoeboid transition (MAT)” [17, 18], characterized by the appearance of plasma membrane blebs, thus providing an explanation for why MMP inhibitors failed to inhibit cancer metastasis in clinical trials [19]. Therefore, in the absence of ECM cues, leader bleb-based migration offers an alternative, integrin and MMP-independent mode of cancer cell migration.

In the current study, we used a targeted screen of oncogenic signaling pathways to identify a pathway with potent effects on MATs. We use BRAF V600E mutant melanoma cells because (1) bleb-based forms of migration have been observed both in vivo and in vitro [12, 15, 20], (2) melanoma often metastasizes non-hematologically, more specifically, not carried to distant tissues by the circulatory system [21], and (3) melanoma has an exceptionally high morbidity rate after it has metastasized. In addition, by combining a simple method for confining cells in vitro and live-cell imaging, we find that the Src pathway differentially has an impact on adhered and blebbing cells. Furthermore, atomic force microscopy (AFM) of non-adherent cells shows that Src activity influences cell mechanical properties in unanticipated ways but is predictive of the likelihood that a cell will undergo leader bleb-based migration.

## Results

### c-Src inhibition induces de-adhesion and plasma membrane blebbing

**Fig. 1:**
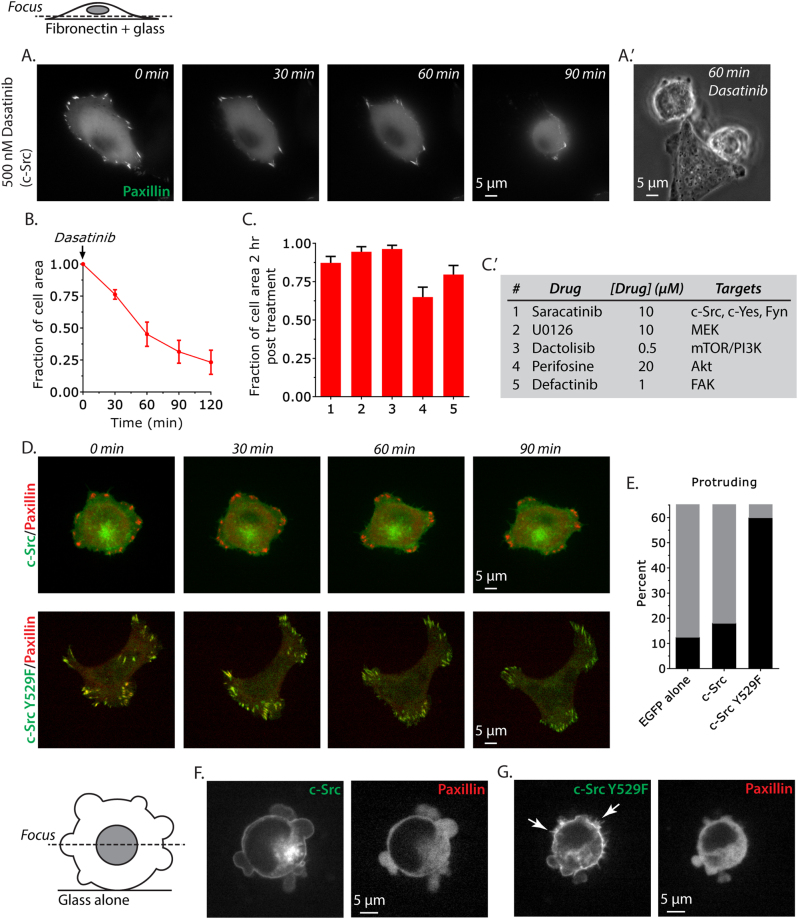
c-Src inhibition induces de-adhesion and plasma membrane blebbing. **a** Widefield time-lapse imaging of the ventral surface of EGFP-paxillin-expressing A375 cells plated on fibronectin-coated glass. Dasatinib was added at “0 min”. **a**' Phase contrast image of Dasatinib-treated, de-adhered, blebbing A375 cells plated on fibronectin-coated glass. **b** Quantitative evaluation of ventral cell surface area after Dasatinib treatment (10 cells). **c** Amalgamated data for all drug treatments (10 cells per condition). **c′** Table of drugs, concentrations used, and their targets in A375 cells. **d** Time-lapse imaging of EGFP-c-Src WT and Y529F with FusionRed-paxillin on fibronectin-coated glass. **e** Percent of protruding (black bars) A375 cells expressing EGFP, EGFP-c-Src, or EGFP-c-Src^Y529F^ was determined from time-lapse imaging over 5 h on fibronectin-coated glass (20 cells per condition). **f** Spinning disk confocal images of the central Z-section of blebbing A375 cells co-expressing EGFP-c-Src and FusionRed-paxillin on uncoated glass. **g** Spinning disk confocal images of the central Z-section of A375 cells co-expressing EGFP-c-Src^Y529F^ and FusionRed-paxillin on uncoated glass. Arrows point to the appearance of protrusions in c-Src^Y529F^-expressing cells. All data are representative of at least two independent experiments

To identify oncogenic signaling pathways that may influence MAT, we undertook a targeted screen using, with the exception of U0126, small molecule kinase inhibitors currently FDA-approved or in clinical trials for the treatment of various cancers. This screen revealed that the Src inhibitor, Dasatinib, currently prescribed for the treatment of chronic myeloid leukemia (CML), could trigger rapid de-adhesion of human BRAF V600E mutant melanoma A375 cells from fibronectin-coated glass, as monitored by the enhanced green fluorescent protein (EGFP)-tagged focal adhesion marker paxillin (Figs. 1a, b and Supplementary Movies 1, 2). Furthermore, de-adhered cells displayed many blebs (Fig. 1a’). Saracatinib, which is a slightly less potent inhibitor of Src [22, 23], had little effect on de-adhesion (Fig. 1c and Supplementary Fig. 1A). Using a Dasatinib-resistant form of Src [24], de-adhesion was shown to require inhibition of Src, as opposed to other targets of Dasatinib (Supplementary Fig. 2). Inhibitors of MEK, the mTOR/PI3K pathway, Akt, and FAK had little effect on de-adhesion (Fig. 1c and Supplementary Fig. 1A).

In light of the above results, we focused on the role of Src in MAT. We first explored what effect Src activity has on A375 cells plated on fibronectin-coated glass. Long-term live-cell imaging of cells expressing EGFP-tagged Src and FusionRed -tagged paxillin showed that expression of Src had little effect on the migration of A375 cells (Fig. 1d, e and Supplementary Movie 3). In fact, we found that human BRAF V600E mutant melanoma A375 cells display very little motility on fibronectin-coated glass (Fig. 1d, e and Supplementary Movie 3). We then expressed EGFP-tagged open conformation and constitutively active, Src^Y529F^, and performed live-cell imaging. In contrast, expressing Src^Y529F^ markedly increased the frequency of protruding and randomly migrating cells, which may be a consequence of changes to actin and focal adhesion dynamics (Figs. 1d, e and Supplementary Movie 4). Interestingly, these cells extended multiple lamellipodia having many focal adhesions at their periphery, with the majority of EGFP-Src^Y529F^ co-localized with FusionRed-paxillin in adhesions (Figs. 1d, e and Supplementary Movie 4).

We next examined what effect Src and Src^Y529F^ expression have on A375 cells plated on uncoated glass, which continuously bleb. In blebbing cells, EGFP-Src localized to the plasma membrane and endomembranes (Fig. 1f and Supplementary Movie 5). As expected, in non-adherent cells FusionRed-paxillin diffusely localized in the cytoplasm (Fig. 1f and Supplementary Movie 5), whereas in blebbing cells the expression of EGFP-Src^Y529F^ induced the formation of many small protrusions (arrows; Fig. 1g and Supplementary Movie 6). In Src WT and Y529F-expressing cells, FusionRed-paxillin diffusely localized in the cytoplasm (Fig. 1g and Supplementary Movie 6). Therefore, high Src activity can promote exploration of adhered cells, whereas in non-adhered blebbing cells it induces protrusion formation.

### High Src activity promotes protrusion formation and decreases blebbing on glass substrates and in 3D collagen matrices

**Fig. 2:**
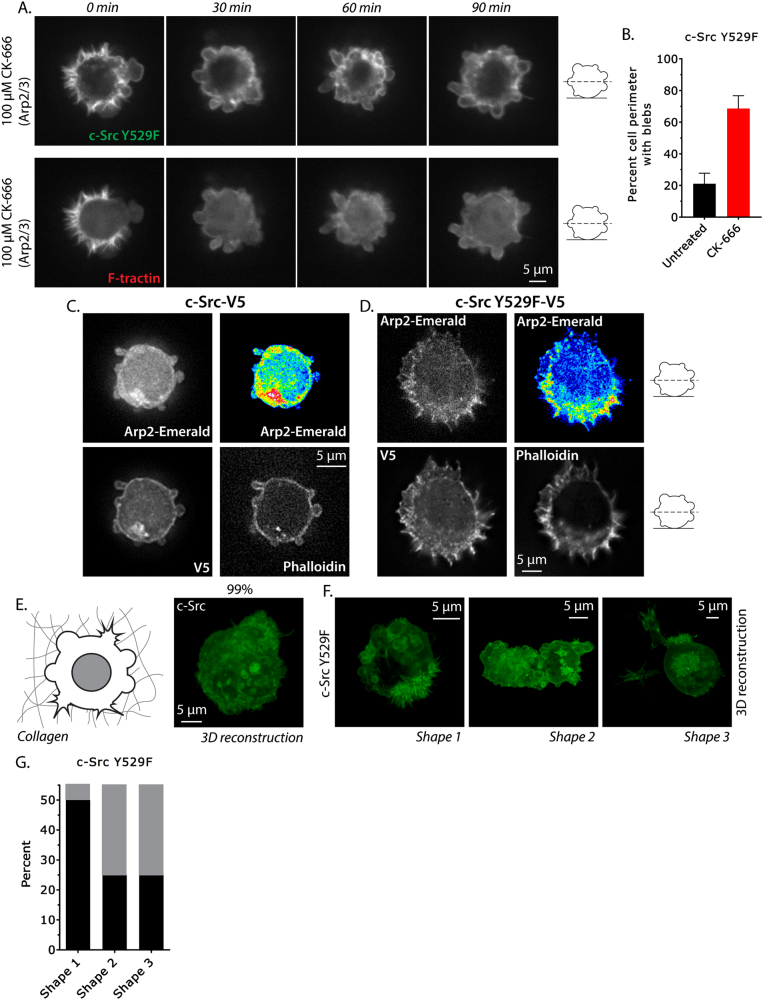
High Src activity promotes protrusion formation and decreases blebbing on glass substrates and in 3D collagen matrices. **a** Spinning disk confocal images of the central Z-section of blebbing A375 cells co-expressing EGFP-c-Src^Y529F^ and FusionRed-F-tractin on uncoated glass. CK-666 was added at “0 min”. **b** Quantitative evaluation of the percent cell perimeter with blebs before or after CK-666 treatment (five cells per condition). **c**,**d** A375 cells freshly plated on poly-L-lysine-coated glass and paraformaldehyde-fixed expressing c-Src-V5 (**c**) and c-Src^Y529F^-V5 (**d**) with Arp2-Emerald. The V5 epitope tag was used to stain for c-Src and fluorescently conjugated phalloidin for F-actin. Pseudocolor of Arp2-Emerald in the upper right quadrant is used to highlight its concentration in cell protrusions (**d**). Images were acquired using a GE DeltaVision Elite microscopy system. **e** 3D reconstruction of a single A375 cell expressing EGFP-c-Src embedded in a 1.5 mg/mL collagen type I gel. Z-stacks were acquired on a Zeiss LSM 880 Airyscan. **f** 3D reconstructions of single A375 cells expressing EGFP-c-Src Y529F embedded in a 1.5 mg/mL collagen type I gel. The three most commonly observed shapes are shown. **g** Percent of Src^Y529F^-expressing cells (*n* = 30) taking on shape 1, 2, or 3. All data are representative of two independent experiments

The aforementioned results are consistent with known substrates of Src, such as cortactin and C3G, which can go on to activate the Arp2/3 complex to promote the formation of branched actin networks [25]. To determine whether the protrusions formed in cells expressing constitutively active Src^Y529F^ depend on Arp2/3, we performed time-lapse imaging of non-adherent, blebbing, A375 cells expressing EGFP-Src^Y529F^ subsequent to the addition of the Arp2/3 inhibitor CK-666. Strikingly, within 30 min after adding CK-666, protrusions disappeared and were replaced by plasma membrane blebs (Fig. 2a, b and Supplementary Movie 7). Additional experiments showed that a lower concentration of CK-666 (20 µM) could inhibit protrusion formation but, interestingly, was insufficient to rescue blebbing (Supplementary Fig. 3A&B). Next, we used Emerald-tagged Arp2 to localize the Arp2/3 complex in Src WT and Y529F-expressing cells freshly plated and fixed on polylysine-coated glass. These experiments revealed that in blebbing cells Arp2 is diffusely localized in the cytoplasm and to specific regions with Src (V5) and F-actin (phalloidin), which are likely to be sites of plasma membrane trafficking (Fig. 2c). In A375 cells expressing Src^Y529F^, however, Arp2 is enriched in protrusions with Src^Y529F^ (V5) and F-actin (phalloidin; Fig. 2d). Collectively, these results are in line with the notion that Src^Y529F^ activates the Arp2/3 complex to drive protrusion formation.

We next set out to determine whether Src^Y529F^ was sufficient to cause cells to protrude in collagen gels. To accomplish this, we embedded A375 cells expressing EGFP-tagged Src WT or Y529F in collagen type I gels (1 mg/mL), and visualized them by super-resolution laser scanning confocal microscopy. This analysis showed that the surface of cells expressing Src WT consistently was covered by plasma membrane blebs (99%), as viewed by three-dimensional (3D) reconstructions (Fig. 2e). Next, we evaluated the effects of Src^Y529F^ on cells embedded in collagen. This revealed that high Src activity could cause a subset of cells to protrude. More specifically, ~50% (*n* = 30 cells) adopted a rounded shape with both blebs and protrusions on its surface (Fig. 2f, g), whereas ~25% took on an elongated shape with both protrusions and blebs (Fig. 2f, g). The remaining ~25% adopted a round shape with no blebs and long, dendritic-like protrusions (Fig. 2f, g), as revealed by 3D reconstructions. It is noteworthy, however, that expression of either Src WT or Y529F was insufficient to induce motility of A375 cells in collagen type I gels. Although considering that collagen density, fiber size, and pore size all affect migration, it is possible that the current conditions are not permissive to this mode of motility. Alternatively, this observation may be considered consistent with the notion that melanomas often use intratumoral lymphatic vessels in order to metastasize [21].

### SrcY529F activates Rac and actin polymerization at the cell periphery

**Fig. 3:**
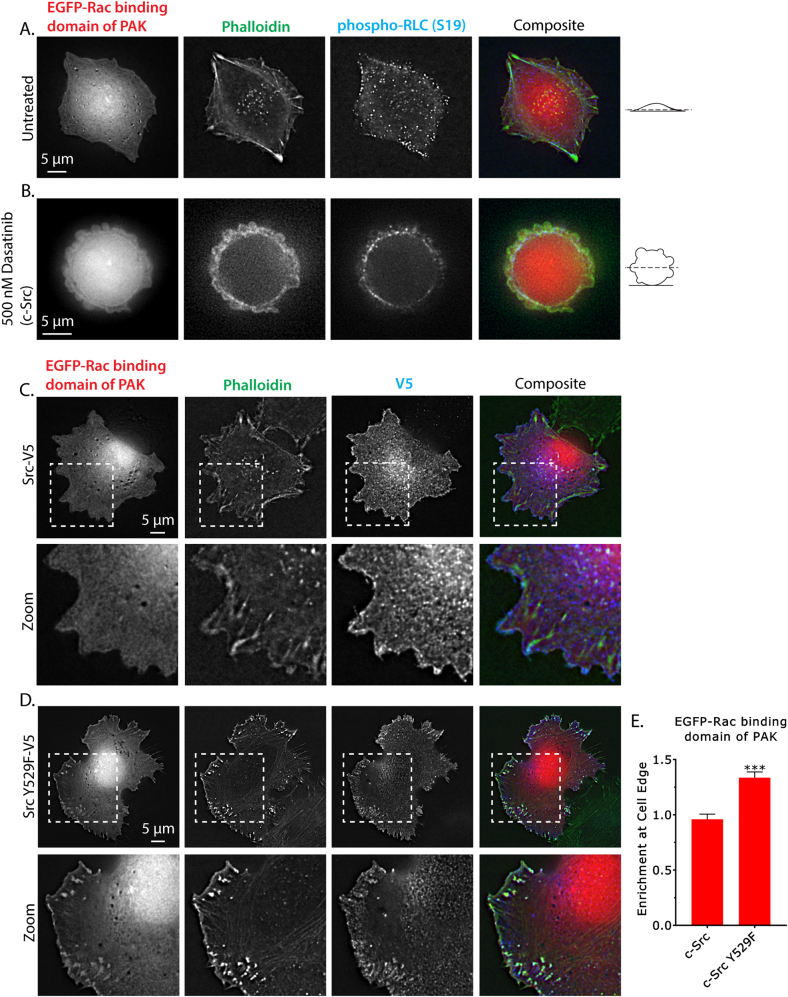
Src^Y529F^ activates Rac and actin polymerization at the cell periphery. **a**,**b** Control (**a**) and 500 nM Dasatinib-treated (90 min; **b**) paraformaldehyde-fixed A375 cells plated on fibronectin-coated glass expressing EGFP-Rac-binding domain of PAK stained for F-actin using fluorescently conjugated phalloidin and phosphorylated (S19) regulatory light chain of myosin (phospho-RLC). **c**,**d** c-Src WT-V5 (**c**) and c-Src Y529F-V5- (**d**) expressing paraformaldehyde-fixed A375 cells plated on fibronectin-coated glass co-expressing EGFP-Rac-binding domain of PAK and stained for F-actin using fluorescently conjugated phalloidin. Images were acquired using a GE DeltaVision Elite microscopy system. All data are representative of two independent experiments. **e** Quantitative evaluation of EGFP-Rac-binding domain of PAK at cell edges (10 cells per condition). “Enrichment at Cell Edge” is the ratio of fluorescence at the cell edge to the cytoplasm. Error is SEM. Statistical significance was determined by a two-tailed Student’s *t*-test. ***p ≤ 0.001

In order to more thoroughly address the possibility that Src^Y529F^ could be indirectly activating Rac and therefore the Arp2/3 complex, adherent A375 cells were transfected with the Rac-binding domain of PAK (EGFP-Rac BD) fused to EGFP, which has been used to mark the location of active Rac in intact cells [26]. In untreated cells, EGFP-Rac BD was uniformly localized in the cytoplasm of transfected A375 cells (Fig. 3a). Using fluorescently conjugated phalloidin, we observed long actin filaments extending inwards and along the cell periphery (Fig. 3a). Staining for phosphorylated (S19) regulatory light chain (RLC) of myosin (phospho-RLC), a marker for active myosin, showed it to be enriched on the longest actin fibers (Fig. 3a). As expected, cells treated with 500 nM Dasatinib (90 min) de-adhered and formed blebs (Fig. 3b). In these cells, EGFP-Rac BD was uniformly localized, phalloidin staining revealed a circumferential actin cortex, and phospho-RLC (S19) staining co-localized with the actin cortex (Fig. 3b). Therefore, in untreated and Dasatinib-treated A375 cells there is no discernable enrichment of active Rac near the cell periphery.

Using EGFP-Rac BD, we determined how the overexpression of V5-tagged c-Src influences Rac activity. To do this, we co-transfected EGFP-Rac BD with c-Src-V5 and stained A375 cells for F-actin (phalloidin) and V5. This showed c-Src-V5 to be enriched at the plasma membrane, long actin filaments extending inwards and along the cell periphery, and EGFP-Rac BD uniformly localized in the cytoplasm (Fig. 3c). Co-transfection of EGFP-Rac BD with the constitutively active form, c-Src Y529F-V5, revealed Src^Y529F^ to be localized on the plasma membrane and within focal adhesions (Fig. 3d). In agreement with previous data, expression of Src^Y529F^ induced fan-like plasma membrane protrusions (Fig. 3d). Strikingly, phalloidin staining revealed a dense F-actin network at the plasma membrane that co-localized with an enrichment of EGFP-Rac BD (Fig. 3d). A quantitative evaluation of EGFP-Rac BD fluorescence at the cell edge relative to the cytoplasm showed that in c-Src-V5-expressing cells this ratio is ~0.95, whereas this ratio is ~1.4 in c-Src^Y529F^-V5-expressing cells (Fig. 3e). Notably, overexpression of dominant-negative Rac^T17N^ could rescue blebbing in Src^Y529F^-expressing cells (Supplementary Fig. 3C-E). Therefore, our data support the hypothesis that Src^Y529F^ will activate Rac and, thus, branched actin filament polymerization at or near the plasma membrane of A375 cells.

### Leader bleb-based migration is resistant to Src inhibition

**Fig. 4:**
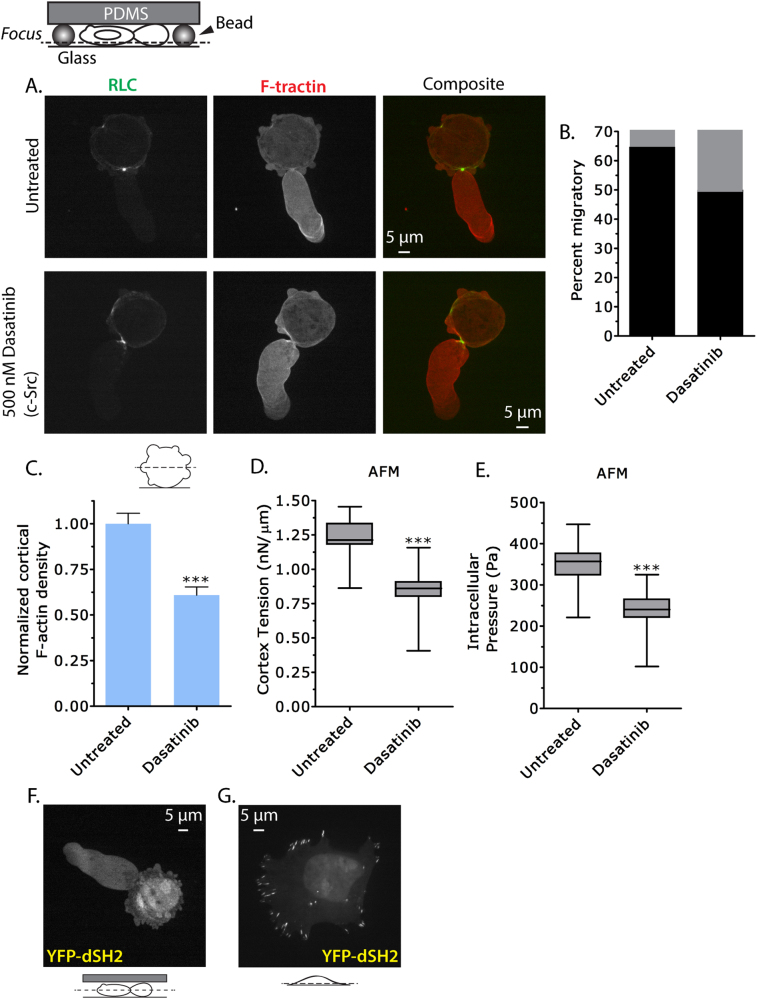
Leader bleb-based migration is resistant to Src inhibition. **a** Ventral Z-section of an EGFP-RLC- and FusionRed-F-tractin-expressing A375 cell treated with 500 nM Dasatinib 90 min prior to being highly confined. **b** Quantitative evaluation of (**a**) for the percent migratory (black bars; leader bleb-based) cells with (*n* = 15) or without (*n* = 38) Dasatinib treatment. **c** Normalized cortical F-actin density in A375 cells with (*n* = 12) or without (*n* = 11) Dasatinib treatment as determined by phalloidin staining of the actin cortex. Fluorescence intensity measurements were measured from the central Z-section of cells freshly plated on poly-L-lysine-coated glass. **d**,**e** Cortex tension and intracellular pressure of A375 cells with (*n* = 27) or without (*n* = 28) Dasatinib treatment. Measurements were taken on freshly plated, round, A375 cells on uncoated glass. Intracellular pressures were calculated from cortical tension and cell radii measured by light microscopy. **f** Central Z-section of a YFP-tagged tandem SH2 domain construct (YFP-dSH2) expressed in a highly confined A375 cell. **g** Ventral Z-section of a YFP-dSH2 expressing A375 cell on fibronectin-coated glass. All data are representative of at least three independent experiments. Statistical significance was determined by one-way ANOVA. ***p ≤ 0.001

To evaluate the role of Src during focal adhesion-independent migration, we used a novel approach to trigger “fast amoeboid” or leader bleb-based migration. This method places cells under a slab of PDMS, which is held at a defined height by micron-sized beads. We use ~3 µm sized beads because this confinement height has been shown to robustly trigger “fast amoeboid” or leader bleb-based migration [12]. To assess the role of Src during leader bleb-based migration, we pretreated A375 cells with Dasatinib for 90 min before subjecting them to high confinement. To visualize F-actin we used FusionRed-tagged F-tractin [15, 27], and for myosin EGFP-tagged RLC. These probes revealed a concentration of ordered cortical actin in leader blebs and myosin highly enriched at the neck separating the leader bleb from the cell body (Fig. 4a). This architecture is consistent with leader bleb-based migration, suggesting that Src inhibition has little effect on this mode of cancer cell migration. Careful examination of many cells showed a relatively small reduction in the percent of cells that form a stable leader bleb (untreated: ~65%, Dasatinib: ~50%; Fig. 4b).

To examine what effect Src has on the cortical actin cytoskeleton of A375 cells, we treated non-adherent blebbing A375 cells with Dasatinib for 90 min prior to fixation and staining with fluorescently conjugated phalloidin, to measure cortical F-actin content. These measurements showed that inhibiting Src could reduce cortical F-actin density by over 35% (Fig. 4c). Because of the likelihood that blebs will be produced often correlates with cell mechanical properties [15], we employed AFM to measure the cortex tension and intracellular pressure of non-adherent cells. This previously described method combines tip-less cantilevers with freshly plated spherical cells to measure global cortex tension [28]. This analysis showed that Dasatinib reduced cortex tension by 32% (Fig. 4d). The intracellular pressure was similarly reduced by Dasatinib treatment (Fig. 4e). Collectively, these results show that Src activity elevates cortical F-actin density and mechanical properties. In spite of this, the percent of cells undergoing leader bleb-based migration is marginally reduced by Dasatinib treatment. Four possible explanations for this result are (1) that the reduction in mechanical properties is too small to significantly have an impact on leader bleb-based migration, (2) the structure of cortical actin is changed in such a way that it overcomes this reduction, (3) the linkages holding the plasma membrane and cortical actin have been weakened, or (4) a combination has circumvented a reduction in mechanical properties.

To better understand why leader bleb-based migration is less effected by Src inhibition, we localized phosphorylated tyrosine residues in cells using a YFP-tagged tandem SH2 domain construct (YFP-dSH2). In highly confined cells, YFP-dSH2 diffusely localized throughout leader blebs, the cell body, and the nucleus (Fig. 4f). In cells adhered to fibronectin-coated glass, YFP-dSH2 was highly enriched in focal adhesions (Fig. 4g). These data indicate that tyrosine-phosphorylated proteins are not highly enriched at specific intracellular locations in cells with leader blebs, as compared to the enrichment of and well-documented role for Src in focal adhesion dynamics.

### Elevated Src activity deleteriously has an impact on leader bleb-based migration

**Fig. 5:**
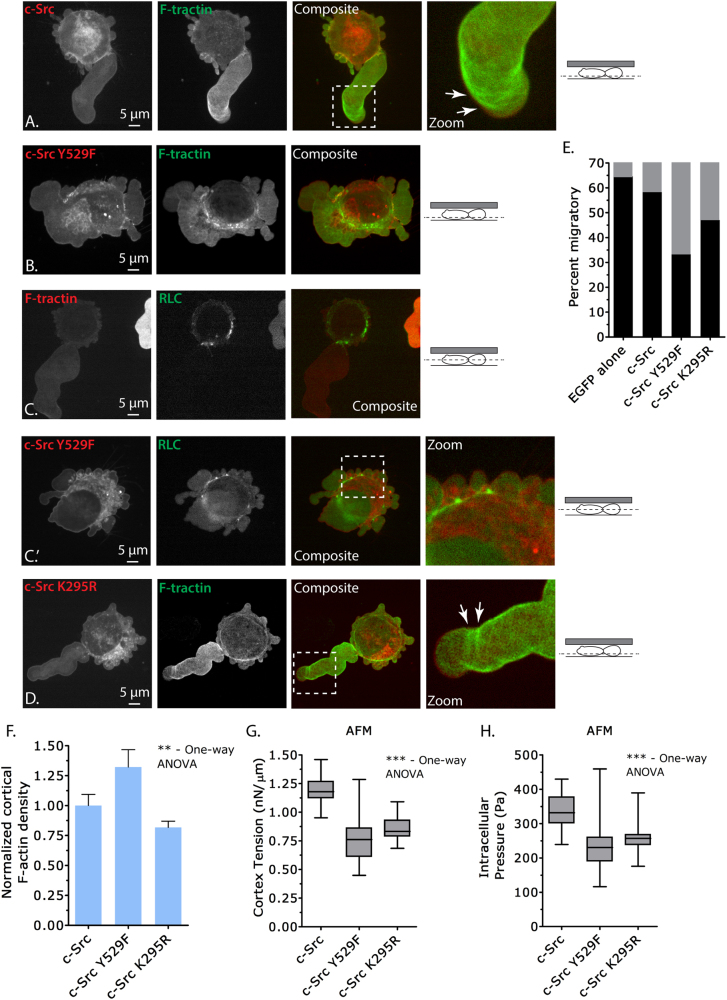
Elevated Src activity deleteriously has an impact on leader bleb-based migration. **a** Ventral Z-section of an EGFP-c-Src- and FusionRed-F-tractin-expressing A375 cell under high confinement. Zoom shows parallel actin fibers near the tip of a large leader bleb. **b** Ventral Z-section of an EGFP-c-Src^Y529F^- and FusionRed-F-tractin-expressing A375 cell under high confinement. **c–c’** Ventral Z-section of either EGFP-RLC and FusionRed-F-tractin (**c**) or EGFP-c-Src^Y529F^- and FusionRed-RLC- (**c’**) expressing A375 cells under high confinement. Zoom shows an uneven distribution of FusionRed-RLC around the cell perimeter of an EGFP-c-Src^Y529F^-expressing cell. **d** Ventral Z-section of an EGFP-c-Src^K295R^- and FusionRed-F-tractin-expressing A375 cell under high confinement. Zoom shows parallel actin fibers near the tip of a large leader bleb. **e** Quantitative evaluation of the percent migratory cells (black bars; leader bleb-based) under high confinement (*n* = 38, 25, 32, and 20 cells). **f** Normalized cortical F-actin density in A375 cells as determined by phalloidin staining of the actin cortex. Fluorescence intensity measurements were measured from the central Z-section of cells freshly plated on poly-L-lysine-coated glass (10 cells per condition). **g**,**h** Cortex tension and intracellular pressure of A375 cells. Measurements were taken on freshly plated, round, A375 cells on uncoated glass (*n* = 21, 25, and 27 cells, respectively). Intracellular pressures were calculated from cortical tension and cell radii measured by light microscopy. All data are representative of at least three independent experiments. Statistical significance was determined by one-way ANOVA. **p ≤ 0.01, ***p ≤ 0.001

To examine the effect of Src activity in more detail during leader bleb-based migration, we expressed EGFP-tagged c-Src in highly confined cells. This revealed that Src localizes to the plasma membrane and endomembranes in cells with leader blebs (Fig. 5a). Furthermore, compared with control “EGFP-alone”-expressing cells, overexpression of Src had little effect on the percent of cells that form stable leader blebs (Fig. 5e and Supplementary Movies 8 and 9). Co-expression of FusionRed-F-tractin showed that cells had parallel actin fibers near the tips of leader blebs (Fig. 5a), which have been previously reported to be important for leader bleb-based migration [15].

Next, we expressed EGFP-tagged open conformation and constitutively active Src^Y529F^ in highly confined cells. Similar to WT Src, Src^Y529F^ was localized to the plasma and endomembranes (Fig. 5a). Strikingly, co-expression of FusionRed-F-tractin revealed in blebs and the cell body a meshwork of actin, as opposed to the parallel actin fibers present near the tips of leader blebs in control cells (Fig. 5b). Furthermore, cells expressing Src^Y529F^ rarely formed large leader blebs (Supplementary Movie 10 and 12). Accordingly, the percent of cells undergoing leader bleb-based migration was significantly reduced by Src^Y529F^ expression (EGFP alone: ~65%, Src^Y529F^: ~32.5%; Fig. 5e). In addition, in Src^Y529F^-expressing cells myosin failed to uniformly decorate the perimeter of the cortex, nor concentrate at the neck of a large leader bleb (Fig. 5c, c’ and Supplementary Movie 11 and 12). Cortical F-actin density was increased by over 25% by the expression of Src^Y529F^ (Fig. 5f). However, cortex tension and intracellular pressure were reduced by 36% and 31%, respectively, in Src^Y529F^-expressing cells (Fig. 5g, h).

We then evaluated the inactive c-Src mutant, Src^K295R^, in highly confined cells. EGFP-tagged Src^K295R^ was, similar to WT Src, localized to the plasma and endomembranes (Fig. 5d). Co-expression of FusionRed-F-tractin showed parallel actin fibers near the tips of large leader blebs (Fig. 5d). Likely because Src^K295R^ functions as dominant-negative, we found a relatively small decrease in the percent of migratory cells (EGFP alone: ~65%, Src^K295R^: ~47.5%; Fig. 5e, Supplementary Movie 13), comparable to the reduction we observed after Dasatinib treatment (untreated: ~65%, Dasatinib: ~50%; Fig. 4b). In addition, cortex tension and intracellular pressure were reduced by 28% and 22%, respectively, in Src^K295R^-expressing cells (Figs. 5g, h). This is comparable to the reduction in mechanical properties observed after Dasatinib treatment (Fig. 4d, e). These results suggest that a heightened level of Src activity, as opposed to the inhibition of, will produce the largest architectural change in cortical actin, inhibiting leader bleb-based migration.

### High Src activity increases the frequency of directionally non-persistent bleb-based migration

**Fig. 6:**
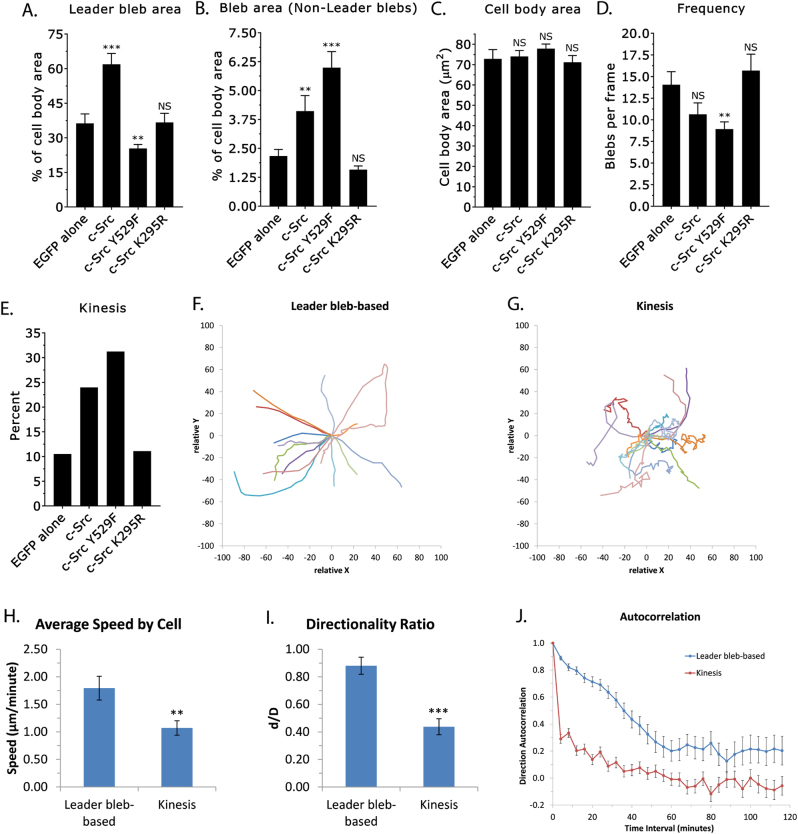
High Src activity increases the frequency of directionally non-persistent bleb-based migration. **a** Quantitative evaluation of, by area, the largest bleb per frame (leader bleb) of highly confined EGFP, c-Src-EGFP, c-Src^Y529F^-EGFP, and c-Src^K295R^-EGFP-expressing A375 cells (*n* = 72 leader blebs (10 cells), 131 leader blebs (15 cells), 101 leader blebs (12 cells), and 130 leader blebs (13 cells), respectively). **b** Quantitation evaluation of bleb area, excluding leader blebs, of highly confined cells (*n* = 196 non-leader blebs (15 cells), 106 non-leader blebs (11 cells), 111 non-leader blebs (14 cells), and 191 non-leader blebs (13 cells), respectively). **c** Quantitative evaluation of cell body area (*n* = 15, 11, 14, and 13 cells, respectively). **d** Quantitative evaluation of blebs per frame (*n* = 15, 11, 14, and 13 cells, respectively). **e** Percent of highly confined cells showing directionally non-persistent migration (black bars; *n* = 38, 25, 32, and 20 cells, respectively). **f–g** Migration tracks for leader bleb-based (**e**) and c-Src^Y529F^-EGFP-promoted directionally non-persistent migration (kinesis; **f**) of highly confined cells (14 cells per condition). **h–j** Output of “DiPer” [29] for average speed by cell (**g**), directionality ratio (**h**), and direction autocorrelation (**I**; 14 cells per condition). All data are representative of at least three independent experiments. Error is SEM. Statistical significance was determined by two-tailed Student’s *t*-tests. **p ≤ 0.01, ***p ≤ 0.001, *NS* not significant

Because cells with high Src activity did show a degree of movement, we carried out an extensive quantitative analysis of bleb dynamics and cell migration. First, we carefully measured the largest single bleb per frame, or leader bleb, in cells expressing EGFP, EGFP-c-Src, EGFP-c-Src^Y529F^, or EGFP-c-Src^K295R^. This analysis revealed that control “EGFP-alone” cells have an average leader bleb area that is ~37.5% of the cell body area (Fig. 6a). Surprisingly, Src overexpressing cells have an average leader bleb area that is ~60% of the cell body area (Fig. 6a). Src^Y529F^-expressing cells have an average leader bleb area that is ~25% of the cell body area (Fig. 6a). However, leader bleb area is unchanged in Src^K295R^-expressing cells (Fig. 6a).

Next, we measured the area of non-leader blebs or all blebs besides the largest single bleb; this analysis showed that the average non-leader bleb area was increased by approximately twofold in Src overexpressing cells (Fig. 6b). The average non-leader bleb area in Src^Y529F^-expressing cells was increased by ~2.75-fold (Fig. 6b). In Src^K295R^-expressing cells, the average non-leader bleb area was decreased by ~1.4-fold; however, this effect was not statistically significant (Fig. 6b). A statistical analysis of cell body area between our conditions confirmed there to be no significant differences (Fig. 6c). As expected, measuring the frequency of blebs, or the average number of blebs per frame, revealed a similar trend. More specifically, EGFP, c-Src-EGFP, c-Src^Y529F^-EGFP, and c-Src^K295R^-EGFP-expressing cells had an average of 14, 11, 9, and 16 blebs per frame, respectively (Fig. 6d).

We next measured the percent of cells that show directionally non-persistent movement, or kinesis. Interestingly, this revealed that kinesis was increased by Src and Src^Y529F^ expression by 2.3-fold and 3-fold, respectively (Fig. 6e). However, the percent of cells that undergo kinesis was unchanged in Src^K295R^-expressing cells (Fig. 6e). Detailed analysis of cell migration tracks by “DiPer” [29] confirmed that on average leader bleb-based migration is more directionally persistent (Fig. 6f, g). Computation of the directionality ratio, defined as the straight-line length between the start point and the end point of the migration trajectory (*d*), divided by the length of the trajectory (*D*), shows that leader bleb-based migration is more directionally persistent (leader bleb-based: 0.88, kinesis: 0.44; Fig. 6i). Because the directionality ratio (*d/D*) is heavily biased by speed [29], we also calculated a direction autocorrelation using “DiPer,” which measures how angles describing the trajectory are aligned with each other [29], confirming a higher degree of directionality for cells undergoing leader bleb-based migration (Fig. 6j). Furthermore, cells overexpressing Src or Src^Y529F^ that undergo kinesis are slower than cells undergoing leader bleb-based migration (leader bleb-based: 1.79 µm/min, kinesis: 1.07 µm/min; Fig. 6h).

Collectively, these analyses have revealed that Src overexpression increases both leader bleb and non-leader bleb areas. In contrast, Src^Y529F^ decreases leader bleb area, but relative to Src overexpression has a greater increase in non-leader bleb area. The increase in non-leader bleb area may account for the greater frequency of directionally non-persistent movement, or kinesis, of cells with high Src activity. In Src-overexpressing cells, the increased leader bleb area may compensate for a simultaneous increase in non-leader bleb area, which can help to preserve the dominating effect of a large stable bleb required for leader bleb-based migration.

### Arp2/3 inhibition rescues leader bleb-based migration in SrcY529F-expressing cells

**Fig. 7:**
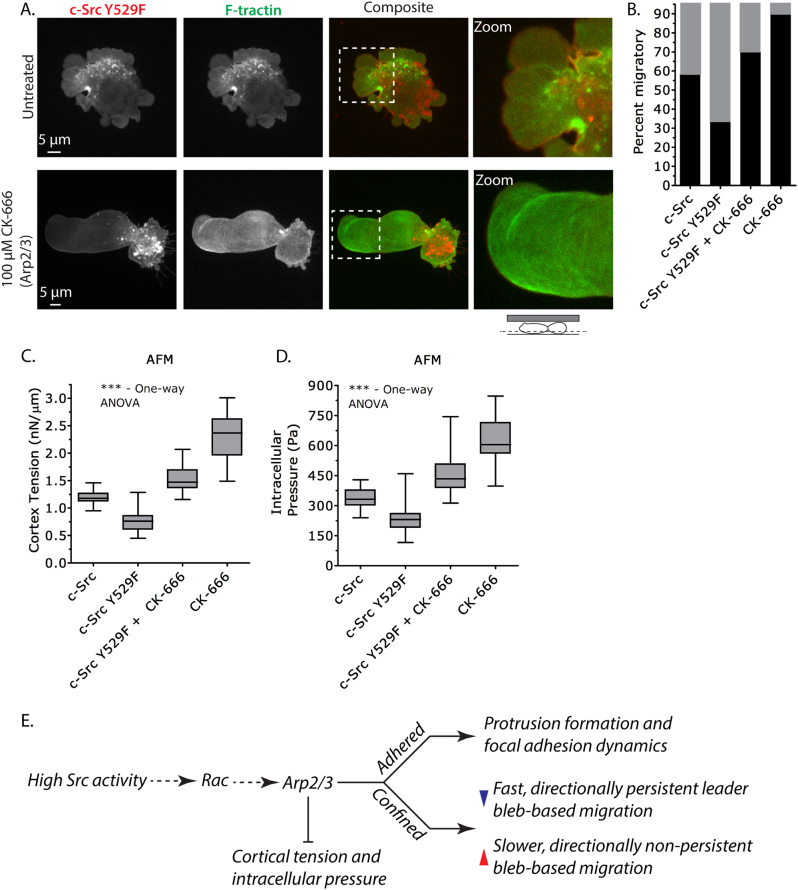
Arp2/3 inhibition rescues leader bleb-based migration in Src^Y529F^-expressing cells. **a** Ventral Z-section of an EGFP-c-Src^Y529F^ and FusionRed-F-tractin-expressing A375 cell treated with 100 µm CK-666 30 min prior to being highly confined. Zoom shows parallel actin fibers near the tip of a large leader bleb. **b** Quantitative evaluation of the percent migratory A375 cells (black bars; leader bleb-based) expressing EGFP-c-Src WT or Y529F with or without CK-666 under high confinement (*n* = 25, 32, 18, and 22 cells, respectively). **c–d** Cortex tension and intracellular pressure of A375 cells. Measurements were taken on freshly plated, round, A375 cells on uncoated glass (*n* = 21, 25, 26, and 23 cells, respectively). Intracellular pressures were calculated from cortical tension and cell radii measured with light microscopy. **e** Schematic depiction of the consequences of high Src activity on cell behavior. All data are representative of at least three independent experiments. Statistical significance was determined by one-way ANOVA. ***p ≤ 0.001

Cortex F-actin density, tension, and intracellular pressure measurements combined with high-resolution live-cell fluorescence microscopy have suggested that high Src activity can decidedly change the architecture of the actin cortex. In support of this hypothesis, Src substrates have been shown to activate the Arp2/3 complex [25], promoting branched actin formation. To directly test whether activation of Arp2/3 by Src inhibits leader bleb-based migration, we incubated Src^Y529F^-expressing A375 cells with a specific inhibitor of Arp2/3, CK-666, before subjecting them to high confinement. Strikingly, CK-666 treatment rescued the formation of large leader blebs in Src^Y529F^-expressing cells (Fig. 7a). More specifically, co-expression of FusionRed-F-tractin showed parallel actin fibers near the tips of large leader blebs (Fig. 7a). Furthermore, incubating Src^Y529F^-expressing cells with CK-666 increased the percent of cells that form stable leader blebs, compared with WT Src controls (Src: ~60%, Src^Y529F^ + CK-666: ~70%; Fig. 7b). Accordingly, we found that cortex tension and intracellular pressure were increased by 29% and 35% over WT Src controls, respectively, in CK-666-treated Src^Y529F^-expressing cells (Fig. 7c, d).

To better understand the impact of Arp2/3-dependent branched actin formation on leader bleb-based migration, we incubated control A375 cells with CK-666 before high confinement. Co-expression of EGFP-RLC and FusionRed-F-tractin showed parallel actin fibers near the tips of very large leader blebs and myosin concentrated near the neck (Supplementary Fig. 4). Careful examination of many CK-666-treated cells revealed a significant increase in the percent of cells forming stable leader blebs (untreated: ~65%, CK-666: ~90%; Fig. 7b). Moreover, cortex tension and intracellular pressure were increased by 90% and 87%, respectively, in CK-666-treated cells (Fig. 7c, d). Therefore, inhibiting Arp2/3 complex activity and, consequently, branched actin formation, potently augments cell mechanical properties and leader bleb-based migration.

## Discussion

The present study was aimed at identifying signaling pathways that may dictate the migration strategy used by BRAF V600E-mutated human melanoma cells. Because melanoma often spreads non-hematologically, to gain access to distant tissues, metastatic melanoma must traverse a diverse array of tissues [21]. This includes collagen matrices, lymphatic capillaries and vessels, basement membranes, perivascular space, and others. Likely, successful metastasis requires that cells adapt their migration mode to the present environment. It has been demonstrated that upon inhibition of integrin engagement or MMPs, cells will undergo a MAT [17, 18], therefore offering an explanation for why MMP inhibitors failed to inhibit metastasis in clinical trials. Characteristic of amoeboid cell migration is the formation of intracellular pressure-driven blebs as opposed to the actin polymerization-driven plasma membrane protrusions typical of mesenchymal motility. More recently, metastatic cancer cells have been shown to respond to physical confinement by switching to “fast amoeboid” [12] or leader bleb-based migration [15]. This type of migration can operate under diverse conditions as it does not require specific integrin-ECM attachments. Therefore, a rational approach for developing inhibitors of metastasis must account for all modes of cancer cell migration.

A targeted screen using clinically useful inhibitors of oncogenic pathways revealed that a specific Src inhibitor, Dasatinib, could induce focal adhesion disassembly and blebbing, which are hallmarks of MAT. It is noteworthy that previous work by others argued that the Src inhibitor, Saracatinib, had no effect on focal adhesion assembly [30]. In this work, suspension cells were treated with Saracatinib and assayed for their ability to adhere. However, in the current study, we treated pre-adhered cells with drug and performed time-lapse imaging for adhesion disassembly. Like these authors, we found that Saracatinib had very little effect on focal adhesion dynamics. The precise nature of the differences between these drugs remains to be determined, but we find that the more potent Src inhibitor, Dasatinib, rapidly induces focal adhesion disassembly. Moreover, using a Dasatinib-resistant form of Src, we found that inhibition of Src was specifically required for focal adhesion disassembly in A375 cells. In support of the role for Src in focal adhesion dynamics, expression of constitutively active, Src^Y529F^, in A375 cells could potently induce cell movement. Furthermore, Src^Y529F^ was found to be highly enriched in focal adhesions.

In blebbing cells, Src was found to be localized to the plasma membrane and endomembranes. Interestingly, expression of Src^Y529F^ in blebbing cells promoted the formation of small, lamellipodia-like, protrusions of the plasma membrane that could be suppressed by dominant-negative Rac^T17N^ and CK-666. In addition, we evaluated the influence of high Src activity on cells inside collagen matrices. Similar to cells on non-adherent substrates, Src^Y529F^ could stimulate protrusion formation. Using a Rac activity reporter, we provided direct evidence that Src^Y529F^ activates Rac, and therefore Arp2/3-dependent branched actin polymerization. We found that, however, treating c-Src^Y529F^-expressing cells with a formin inhibitor diminishes the spiky appearance of these protrusions (Supplementary Fig. 6); therefore, similar to at the edge of canonical migrating cells they contain a mixture of actin filament-based structures. In confined cells with high Src activity, however, our data are in line with the notion that an increase in Arp2/3 activity is responsible for changes in actin and myosin, bleb dynamics, migration velocity, and directional persistence. To mimic the confines of tissues, we used a novel approach that places cells under a slab of PDMS, which is held at a defined height by micron-sized beads. We showed that the assay robustly triggers leader bleb-based migration. Importantly, because the height is determined by the beads used, the effect of confinement level can be evaluated, as it pertains to the specific hypothesis being tested.

Using this new methodology, we found that inhibiting Src activity with Dasatinib has very little effect on the percent of cells undergoing leader bleb-based migration. Despite, a statistically significant decrease in cortical F-actin density and mechanical properties in Dasatinib-treated cells. These results suggest that the aforementioned effects can be circumvented by, for example, a change in cortical actin architecture, a weakening of the linkages that attach the plasma membrane to the actin cortex, myosin activity, or a combination. The most straightforward explanation would be that the decrease in cortical F-actin density, after Dasatinib treatment, weakens the cortex by reducing the amount of actin available for proteins such as Ezrin to bind and link cortical actin to the plasma membrane. Therefore, making it more likely a bleb will form and circumvent a reduction in mechanical properties. Because we observed a marked increase of branched actin in Src^Y529F^-expressing cells, which coincided with a decrease in the percent of cells that form stable leader blebs, it can suggest that a change in cortical actin architecture can also influence the likelihood that a large bleb will form. Interestingly, the localization of myosin was changed by high Src activity in pre-leader bleb cells; myosin is localized uniformly around the cortex, whereas in Src^Y529F^-expressing cells myosin was localized to patches of the cortex. Perhaps, interacting with regions where there is a higher concentration of unbranched formin generated actin that myosin prefers. The uneven association of myosin with the cortex would be predicted to decrease its ability to reduce cell volume, and therefore increase intracellular pressure. In agreement with this view, cell mechanical properties were found to be decreased by Src^Y529F^ expression. Interestingly, high Src activity did increase directionally non-persistent bleb-based movement. These observations are depicted schematically in Fig. 7e. This type of movement appears to be supported by an increase in the area of non-leader blebs. To directly test whether a change of cortical actin architecture was responsible, we treated Src^Y529F^-expressing cells with CK-666. This resulted in mechanical properties being rescued in Src^Y529F^-expressing cells, as well as leader bleb-based migration. In point of fact, CK-666 treatment resulted in values for mechanical properties and the percent of migratory cells that exceeded those of control (Supplementary Fig. 5).

Collectively, our data indicate that Src positively effects, on adherent surfaces, focal adhesion formation and dynamics. In non-adhered, highly confined cells, high Src activity promoted a directionally non-persistent bleb-based movement, whereas inhibiting Src activity has little effect on leader bleb-based migration. This is noteworthy because an inhibitor of focal adhesion-based migration may not necessarily be effective toward other modes of cancer cell motility. It has been proposed that Src inhibitors be evaluated for their potential as cancer metastasis blockers. This idea has been supported by preclinical data, which has shown for example that metastasis could be reduced by Dasatinib and Saracatinib if given early and continuously in model systems [31]. In the clinic, however, progression-free survival in the metastatic setting for several cancers [32–39], including melanoma [40, 41], was not significantly impacted by Dasatinib or Saracatinib. Saracatinib’s development was discontinued by its manufacturer, whereas Dasatinib was approved for the treatment of CML owing to its ability to inhibit the BCR-Abl fusion protein. On the basis of the present study and others, we recommend that preclinical studies of metastasis blockers include model systems for adhesion-independent motility, such as leader bleb-based cancer cell migration.

## Methods

### Cell culture and transfection

A375 cells were obtained from American Type Culture Collection (cat #: CRL-1619, ATCC, Manassas, VA) and maintained for up to 15 passages in DMEM supplemented with 10% FBS (cat #: A3160401, Life Technologies, Carlsbad, CA), GlutaMAX (Life Technologies), antibiotic–antimycotic (Life Technologies), and 20 mM HEPES pH 7.4. A Nucleofector 2b device (Kit V; Lonza, Basel, Switzerland) was used to transfect plasmids. Cells were plated on six-well glass-bottom plates (Cellvis, Mountain View, CA) either directly, after coating with 10 µg/mL human plasma fibronectin (cat #: FC010, Millipore, Billerica, MA), or with poly-L-Lysine (cat #: A-005-C, Millipore), as noted in each figure.

### Drug treatments

U0126 (cat #: 9903), Dactolisib (NVP-BEZ235; cat #: 13101), and Perifosine (cat #: 14240) were purchased from Cell Signaling Technology (Beverly, MA). Dasatinib (cat #: S1021), Saracatinib (cat #: S1006), and Defactinib (cat #: S7654) were purchased from Selleckchem (Houston, TX). CK-666 (cat #: 3950) and SMIFH2 (cat #: 4401) were purchased from Tocris Bioscience (Bristol, UK). To inhibit a pathway, a 1000× stock solution in dimethyl sulfoxide (DMSO; Sigma-Aldrich, St. Louis, MO) was diluted in 400 µL media and dissolved using a vortex mixer for 30 s before adding to wells containing 2 mL of media. For AFM analyses, cells were treated for 30 min prior. Treatments for fluorescence microscopy are as noted in each figure.

### Plasmids

mEmerald-paxillin, EGFP-c-Src, EGFP-c-Src^K295R^ was a kind gift from Michael Davidson (Florida State University). Src^Y529F^ was generated using the QuickChange II XL site-directed mutagenesis kit (Agilent Technologies, Santa Clara, CA) using the following primers:

forward-CTCGACAGAGCCCCAGTTCCAGCCTGGAGAGAAC

reverse-GTTCTCTCCAGGCTGGAACTGGGGCTCTGTCGAG.

Src^T338I^ was generated using the QuickChange II XL site-directed mutagenesis kit using the following primers:

forward-CCCATCTACATCGTCATTGAGTACATGAGC

reverse-GCTCATGTACTCAATGACGATGTAGATGGG

c-Src-V5 and c-Src^Y529F^-V5 were generated by directional TOPO cloning into pcDNA 3.1D-TOPO/V5-His (Life Technologies). FusionRed-F-tractin has been previously described [15]. FusionRed-paxillin, FusionRed-RLC, and farnesylated FusionRed were purchased from Evrogen (Russia). EGFP-tagged Rac-binding domain of PAK (Plasmid #26735; Addgene, Cambridge, MA) was a gift from William Bement (University of Wisconsin). Arp2-Emerald (Plasmid #53993; Addgene) was a gift from Michael Davidson (Florida State University). YFP-dSH2 was a kind gift from Ana Pasapera Limon (NHLBI).

### Microscopy

Imaging of mesenchymal, amoeboid-like, and cells undergoing leader bleb-based migration was performed using a spinning disk confocal system that has been previously described [42].

### Immunofluorescence

Adherent cells were fixed using 4% paraformaldehyde (Electron Microscopy Sciences, Hatfield, PA) in HEPES buffered saline (HBS) for 20 min at room temperature before blocking with HBS containing 1% BSA, 1% fish gelatin, and 0.1% Triton X-100 for 1 h at room temperature. Antibodies for staining phospho-RLC (S19; cat #: out of production, Santa Cruz Biotechnology, Dallas, TX) and V5 (cat #: R960-25, Life Technologies) were used at 1:500 and 1:1000, respectively, in blocking buffer for 2 h at room temperature. After washing with HBS, Alexa Fluor 568-conjugated secondary’s (Life Technologies) were used at 1:400 in blocking buffer for 2 h at room temperature. Alexa Fluor 647-conjugated phalloidin (Life Technologies) was used at 1:50 in blocking buffer for 2 h at room temperature. After washing, imaging was performed in HBS on a DeltaVision Elite (GE, Fairfield, CT) microscopy system equipped with a ×60 oil objective (1.42 numerical aperture (NA)) and proprietary deconvolution software.

### Confinement under PDMS

Cells were confined under a slab of PDMS, which was held at a defined height. This protocol will be described in detail elsewhere. Briefly, PDMS (Dow Corning 184 SYLGARD) was purchased from Krayden (Westminster, CO). Two milliliters were cured overnight at 37 °C in each well of a six-well glass-bottom plate (Cellvis). Using a scalpel, a 1 cm × 1 cm square was then cut and 3 mL of serum-free media containing 1% BSA was then added to each well and incubated overnight at 37 °C. After aspirating away the serum-free media containing 1% BSA, 200 µL of complete media containing trypsinized cells (250,000 to 1 million) and 2 µL of beads (3.11 µm; Bangs Laboratories, Fishers, IN) was then pipetted into the square opening. The vacuum created by briefly lifting one side of the square opening with a 1 mL pipette tip was used to move cells and beads underneath the PDMS. Finally, 3 mL of complete media was added to each well and cells were recovered in a tissue culture incubator for 30–60 min before imaging.

### Fraction of cell area and percent protruding measurements

Related to Figure 1. To calculate the fraction of cell area after drug treatments, cells were plated on six-well glass-bottom plates (Cellvis) pre-coated overnight at 4 °C with 10 µg/mL human plasma fibronectin (cat #: FC010). Prior to time-lapse imaging, cells were given 2 h to adhere in a tissue culture incubator. Drugs were pre-diluted in 400 µL of media before adding at “0 min” to wells containing 2 mL of media. Cell area was measured in Fiji (http://fiji.sc/Fiji) using the free-hand circle tool at the time points indicated in each figure. “Fraction of cell area” was calculated in Microsoft Excel (Redmond, WA) as the measured area divided by the area at time 0. For percent protruding measurements, cells were similarly plated on fibronectin-coated glass-bottom plates and adhered for 2 h before imaging. Cells were then imaged at 2 min intervals for 5 h by spinning disk confocal. Cells that extended lamellipodia for at least five successive frames were classified as “protruding.” The fraction of cells protruding was calculated in Microsoft Excel, and all results were plotted using GraphPad Prism (San Diego, CA).

### Percent cell perimeter with blebs and collagen-embedded cell shape classification

Related to Figure 2. For calculating the percent cell perimeter with blebs, cells were freshly plated on uncoated glass and imaged through the central Z-section at the time points indicated in the figure and Supplementary Movie 7. The cell perimeter was measured using the free-hand line tool in Fiji, and the percent of the total perimeter having blebs was calculated in Microsoft Excel. Statistical analyses and plots were generated using GraphPad Prism. To classify collagen-embedded cell shapes, collagen matrices were made using rat tail collagen type I (cat #: 08-115, Millipore) following the protocol by Artym and Matsumoto, 2011 [43]. Cells were imaged in collagen matrices on a Zeiss LSM 880 Airyscan (Germany) equipped with a stage-top incubator (Tokai Hit, Japan). 3D reconstructions were made using the “3D Viewer” plugin [44] in Fiji from Z-stacks acquired in Airyscan mode. A thematic analysis for cell morphology was done for 60 cells by eye or confocal. Subsequently, EGFP-c-Src^Y529F^-expressing cells were classified as one of three shapes. All data were plotted using GraphPad Prism.

### Enrichment at cell edge and cortical F-actin density measurements

Related to Figures 3, 4, 5, and 7. To determine the “Enrichment at Cell Edge,” we traced with a 5-pixel wide line the cell edges and measured the mean fluorescence intensity using Fiji. The free-hand circle tool was used to measure the mean fluorescence intensity in the cytoplasm and the “Enrichment at the Cell Edge” was calculated as the ratio of cell edge to cytoplasmic fluorescence in Microsoft Excel. Statistical analyses and plots were generated using GraphPad Prism. For measuring F-actin cortex density, freshly plated, round cells, on poly-L-Lysine-coated glass were fixed using 4% paraformaldehyde (Electron Microscopy Sciences) in HBS for 20 min at room temperature before staining with Alexa Fluor-conjugated phalloidin (Life Technologies) at 1:250 for 1 h. Samples were washed very gently four times before acquiring spinning disk confocal images (×100, 1.4 NA) through the central Z-plane of the cell. Subsequently, a 5-pixel wide line was drawn along a region of the cortex that was free of blebs and the mean fluorescence intensity was measured using Fiji. Background fluorescence was measured by selecting a region outside the cell. F-actin cortex density was then calculated as the mean fluorescence intensity at the cortex minus background fluorescence. Statistical analyses and plots were generated using GraphPad Prism.

### Bleb and cell body areas, frequency, and percent migratory

Related to Figures 4, 5, 6, 7, and Supplementary Fig. 5. For leader bleb, non-leader bleb, and cell body areas, cells freshly confined under PDMS with 3 µm beads were traced using spinning disk confocal images with the free-hand circle tool in Fiji at 30 min intervals for 5 h. For each frame, the percent cell body area for leader or non-leader blebs was calculated in Microsoft Excel. To calculate the frequency or blebs per frame, the total number of blebs was determined every 30 min for 5 h. Statistical analyses and plots were generated using GraphPad Prism. For percent migratory measurements, a cell was defined as migratory if it traveled at least one cell length every hour. Phase contrast images were used to confirm that beads were not obstructing the path of a cell. All data were plotted using GraphPad Prism.

### “DiPer” analyses

Related to Figure 6. To perfom “DiPer” analyses, a plugin for Microsoft Excel developed by Gorelik and colleagues, cells migrating under PDMS with 3 µm beads were manually tracked with the Fiji plugin “MTrackJ” [45] from time-lapse images taken every 2 min for 5 h. For minimizing positional error between frames, we tracked cells every 4 min for 5 h. Phase contrast images were used to confirm that beads were not obstructing the path of a cell. Statistical analyses and plots were generated using Microsoft Excel.

### Atomic force microscopy

Related to Figures 4, 5, 7, and Supplementary Fig. 5. The method used for performing AFM and equations for calculating cortical tension and intracellular pressure are described in detail elsewhere [28].

### Statistics

All sample sizes were empirically determined based on saturation and any outliers were identified by a Grubb’s test using GraphPad QuickCalcs (https://www.graphpad.com/quickcalcs/) and excluded from further analyses. A statistical difference between two conditions was determined using a two-tailed Student’s *t*-test or between two or more samples using a one-way ANOVA in GraphPad Prism. *p ≤ 0.05, **p ≤ 0.01, and ***p ≤ 0.001

## Electronic supplementary material

S1

S2

S3

S4

S5

S6

Movie S1

Movie S2

Movie S3

Movie S4

Movie S5

Movie S6

Movie S7

Movie S8

Movie S9

Movie S10

Movie S11

Movie S12

Movie S13

Supplementary Figure and Movie Legends

## References

[1] Case LB, Waterman CM (2015). Integration of actin dynamics and cell adhesion by a three-dimensional, mechanosensitive molecular clutch. Nat Cell Biol.

[2] Kanchanawong P, Shtengel G, Pasapera AM, Ramko EB, Davidson MW, Hess HF (2010). Nanoscale architecture of integrin-based cell adhesions. Nature.

[3] Plotnikov SV, Pasapera AM, Sabass B, Waterman CM (2012). Force fluctuations within focal adhesions mediate ECM-rigidity sensing to guide directed cell migration. Cell.

[4] Mitra SK, Hanson DA, Schlaepfer DD (2005). Focal adhesion kinase: in command and control of cell motility. Nat Rev Mol Cell Biol.

[5] Schaller MD (2010). Cellular functions of FAK kinases: insight into molecular mechanisms and novel functions. J Cell Sci.

[6] Chan KT, Bennin DA, Huttenlocher A (2010). Regulation of adhesion dynamics by calpain-mediated proteolysis of focal adhesion kinase (FAK). J Biol Chem.

[7] Ezratty EJ, Partridge MA, Gundersen GG (2005). Microtubule-induced focal adhesion disassembly is mediated by dynamin and focal adhesion kinase. Nat Cell Biol.

[8] Ilic D, Furuta Y, Kanazawa S, Takeda N, Sobue K, Nakatsuji N (1995). Reduced cell motility and enhanced focal adhesion contact formation in cells from FAK-deficient mice. Nature.

[9] Webb DJ, Donais K, Whitmore LA, Thomas SM, Turner CE, Parsons JT (2004). FAK-Src signalling through paxillin, ERK and MLCK regulates adhesion disassembly. Nat Cell Biol.

[10] Zhang S, Yu D (2012). Targeting Src family kinases in anti-cancer therapies: turning promise into triumph. Trends Pharmacol Sci.

[11] Bergert M, Erzberger A, Desai RA, Aspalter IM, Oates AC, Charras G (2015). Force transmission during adhesion-independent migration. Nat Cell Biol.

[12] Liu YJ, Le Berre M, Lautenschlaeger F, Maiuri P, Callan-Jones A, Heuze M (2015). Confinement and low adhesion induce fast amoeboid migration of slow mesenchymal cells. Cell.

[13] Klemke RL, Cai S, Giannini AL, Gallagher PJ, de Lanerolle P, Cheresh DA (1997). Regulation of cell motility by mitogen-activated protein kinase. J Cell Biol.

[14] Charras GT, Yarrow JC, Horton MA, Mahadevan L, Mitchison TJ (2005). Non-equilibration of hydrostatic pressure in blebbing cells. Nature.

[15] Logue JS, Cartagena-Rivera AX, Baird MA, Davidson MW, Chadwick RS, Waterman CM (2015). Erk regulation of actin capping and bundling by Eps8 promotes cortex tension and leader bleb-based migration. Elife.

[16] Ruprecht V, Wieser S, Callan-Jones A, Smutny M, Morita H, Sako K (2015). Cortical contractility triggers a stochastic switch to fast amoeboid cell motility. Cell.

[17] Sahai E, Marshall CJ (2003). Differing modes of tumour cell invasion have distinct requirements for Rho/ROCK signalling and extracellular proteolysis. Nat Cell Biol.

[18] Wolf K, Mazo I, Leung H, Engelke K, von Andrian UH, Deryugina EI (2003). Compensation mechanism in tumor cell migration: mesenchymal-amoeboid transition after blocking of pericellular proteolysis. J Cell Biol.

[19] Coussens LM, Fingleton B, Matrisian LM (2002). Matrix metalloproteinase inhibitors and cancer: trials and tribulations. Science.

[20] Tozluoglu M, Tournier AL, Jenkins RP, Hooper S, Bates PA, Sahai E (2013). Matrix geometry determines optimal cancer cell migration strategy and modulates response to interventions. Nat Cell Biol.

[21] Wong SY, Hynes RO (2006). Lymphatic or hematogenous dissemination: how does a metastatic tumor cell decide?. Cell Cycle.

[22] Green TP, Fennell M, Whittaker R, Curwen J, Jacobs V, Allen J (2009). Preclinical anticancer activity of the potent, oral Src inhibitor AZD0530. Mol Oncol.

[23] O’Hare T, Walters DK, Stoffregen EP, Jia T, Manley PW, Mestan J (2005). In vitro activity of Bcr-Abl inhibitors AMN107 and BMS-354825 against clinically relevant imatinib-resistant Abl kinase domain mutants. Cancer Res.

[24] Lombardo LJ, Lee FY, Chen P, Norris D, Barrish JC, Behnia K (2004). Discovery of N-(2-chloro-6-methyl-phenyl)-2-(6-(4-(2-hydroxyethyl)-piperazin-1-yl)-2-methylpyrimidin-4-ylamino) thiazole-5-carboxamide (BMS-354825), a dual Src/Abl kinase inhibitor with potent antitumor activity in preclinical assays. J Med Chem.

[25] Ferrando IM, Chaerkady R, Zhong J, Molina H, Jacob HK, Herbst-Robinson K (2012). Identification of targets of c-Src tyrosine kinase by chemical complementation and phosphoproteomics. Mol Cell Proteom.

[26] Benink HA, Bement WM (2005). Concentric zones of active RhoA and Cdc42 around single cell wounds. J Cell Biol.

[27] Schell MJ, Erneux C, Irvine RF (2001). Inositol 1,4,5-trisphosphate 3-kinase A associates with F-actin and dendritic spines via its N terminus. J Biol Chem.

[28] Cartagena-Rivera AX, Logue JS, Waterman CM, Chadwick RS (2016). Actomyosin cortical mechanical properties in nonadherent cells determined by atomic force microscopy. Biophys J.

[29] Gorelik R, Gautreau A (2014). Quantitative and unbiased analysis of directional persistence in cell migration. Nat Protoc.

[30] Horton ER, Humphries JD, Stutchbury B, Jacquemet G, Ballestrem C, Barry ST (2016). Modulation of FAK and Src adhesion signaling occurs independently of adhesion complex composition. J Cell Biol.

[31] Kim LC, Song L, Haura EB (2009). Src kinases as therapeutic targets for cancer. Nat Rev Clin Oncol.

[32] Finn RS, Bengala C, Ibrahim N, Roche H, Sparano J, Strauss LC (2011). Dasatinib as a single agent in triple-negative breast cancer: results of an open-label phase 2 study. Clin Cancer Res.

[33] Fury MG, Baxi S, Shen R, Kelly KW, Lipson BL, Carlson D (2011). Phase II study of saracatinib (AZD0530) for patients with recurrent or metastatic head and neck squamous cell carcinoma (HNSCC). Anticancer Res.

[34] Gucalp A, Sparano JA, Caravelli J, Santamauro J, Patil S, Abbruzzi A (2011). Phase II trial of saracatinib (AZD0530), an oral SRC-inhibitor for the treatment of patients with hormone receptor-negative metastatic breast cancer. Clin Breast Cancer.

[35] Mackay HJ, Au HJ, McWhirter E, Alcindor T, Jarvi A, MacAlpine K (2012). A phase II trial of the Src kinase inhibitor saracatinib (AZD0530) in patients with metastatic or locally advanced gastric or gastro esophageal junction (GEJ) adenocarcinoma: a trial of the PMH phase II consortium. Invest New Drugs.

[36] Mayer EL, Baurain JF, Sparano J, Strauss L, Campone M, Fumoleau P (2011). A phase 2 trial of dasatinib in patients with advanced HER2-positive and/or hormone receptor-positive breast cancer. Clin Cancer Res.

[37] Molina JR, Foster NR, Reungwetwattana T, Nelson GD, Grainger AV, Steen PD (2014). A phase II trial of the Src-kinase inhibitor saracatinib after four cycles of chemotherapy for patients with extensive stage small cell lung cancer: NCCTG trial N-0621. Lung Cancer.

[38] Schilder RJ, Brady WE, Lankes HA, Fiorica JV, Shahin MS, Zhou XC (2012). Phase II evaluation of dasatinib in the treatment of recurrent or persistent epithelial ovarian or primary peritoneal carcinoma: a Gynecologic Oncology Group study. Gynecol Oncol.

[39] Sharma MR, Wroblewski K, Polite BN, Knost JA, Wallace JA, Modi S (2012). Dasatinib in previously treated metastatic colorectal cancer: a phase II trial of the University of Chicago Phase II Consortium. Invest New Drugs.

[40] Gangadhar TC, Clark JI, Karrison T, Gajewski TF (2013). Phase II study of the Src kinase inhibitor saracatinib (AZD0530) in metastatic melanoma. Invest New Drugs.

[41] Kluger HM, Dudek AZ, McCann C, Ritacco J, Southard N, Jilaveanu LB (2011). A phase 2 trial of dasatinib in advanced melanoma. Cancer.

[42] Shin WD, Fischer RS, Kanchawong P, Kim Y, Lim J, Myers KA, Nishimura Y, Plotnikov SV, Thievessen I, Yarar D, Sabass B, Waterman CM. A versatile, multicolor total internal reflection fluorescence and spinning-disk confocal microscope system for high-resolution live cell imaging. In: Goldman RD SJ, Spector DL, editors. Live cell imaging: a laboratory manual. 2nd ed*.* Cold Spring Harbor: Cold Spring Harbor Laboratory Press; 2010. p. 119–38.

[43] Artym VV, Matsumoto K (2010). Imaging cells in three-dimensional collagen matrix. Curr Protoc Cell Biol.

[44] Schmid B, Schindelin J, Cardona A, Longair M, Heisenberg M (2010). A high-level 3D visualization API for Java and Image. J BMC Bioinformation.

[45] Meijering E, Dzyubachyk O, Smal I (2012). Methods for cell and particle tracking. Methods Enzymol.

