# Supplementary figures and images for: c-Src activity is differentially required by cancer cell motility modes
Source: Oncogene. 2018 Jan 30;37(16):2104–21. doi: 10.1038/s41388-017-0071-5 (PMC5906457; doi:10.1038/s41388-017-0071-5)

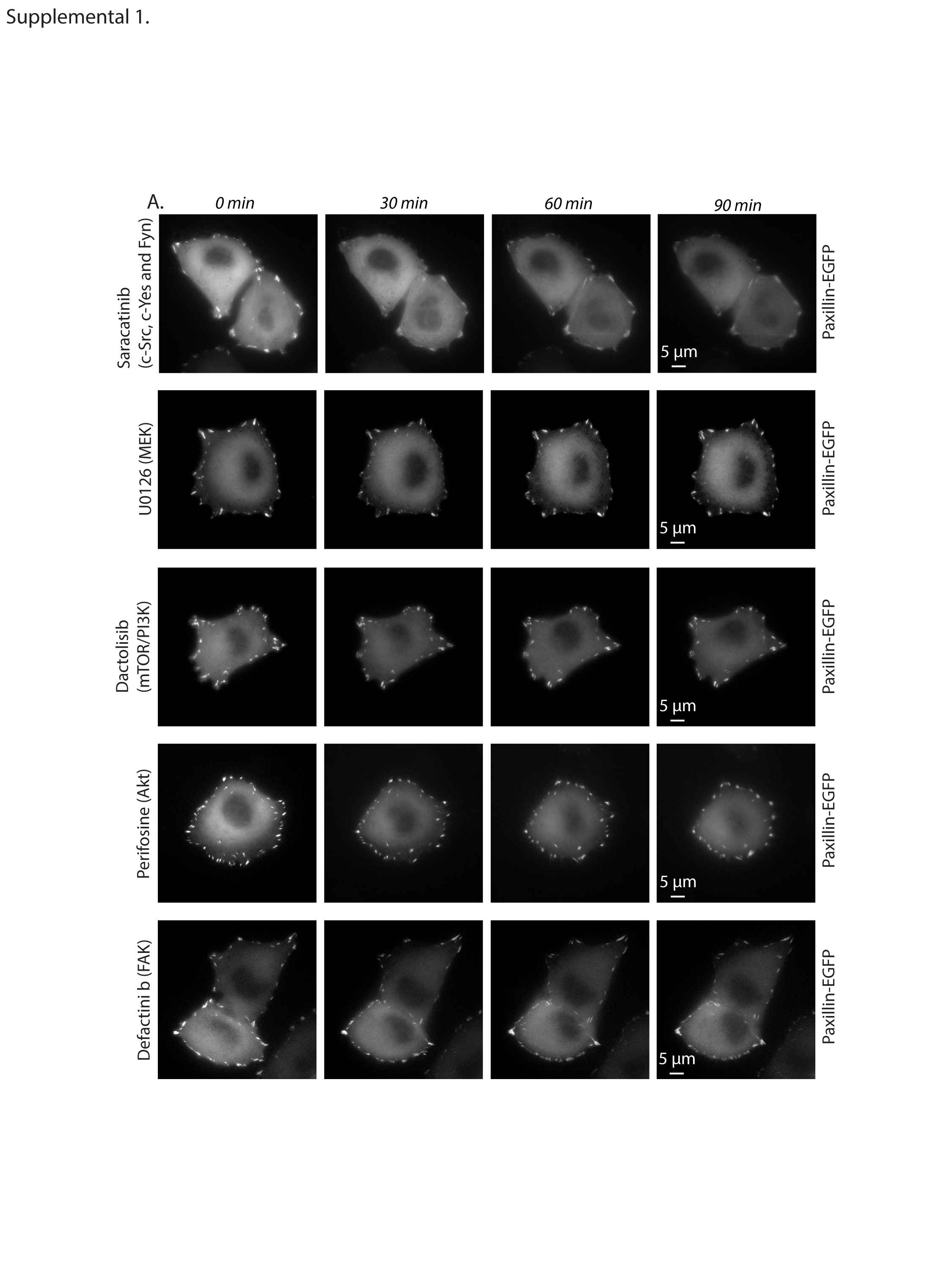

Supplement: Supplementary file 1 — S1 [file 41388_2017_71_MOESM1_ESM.tif]

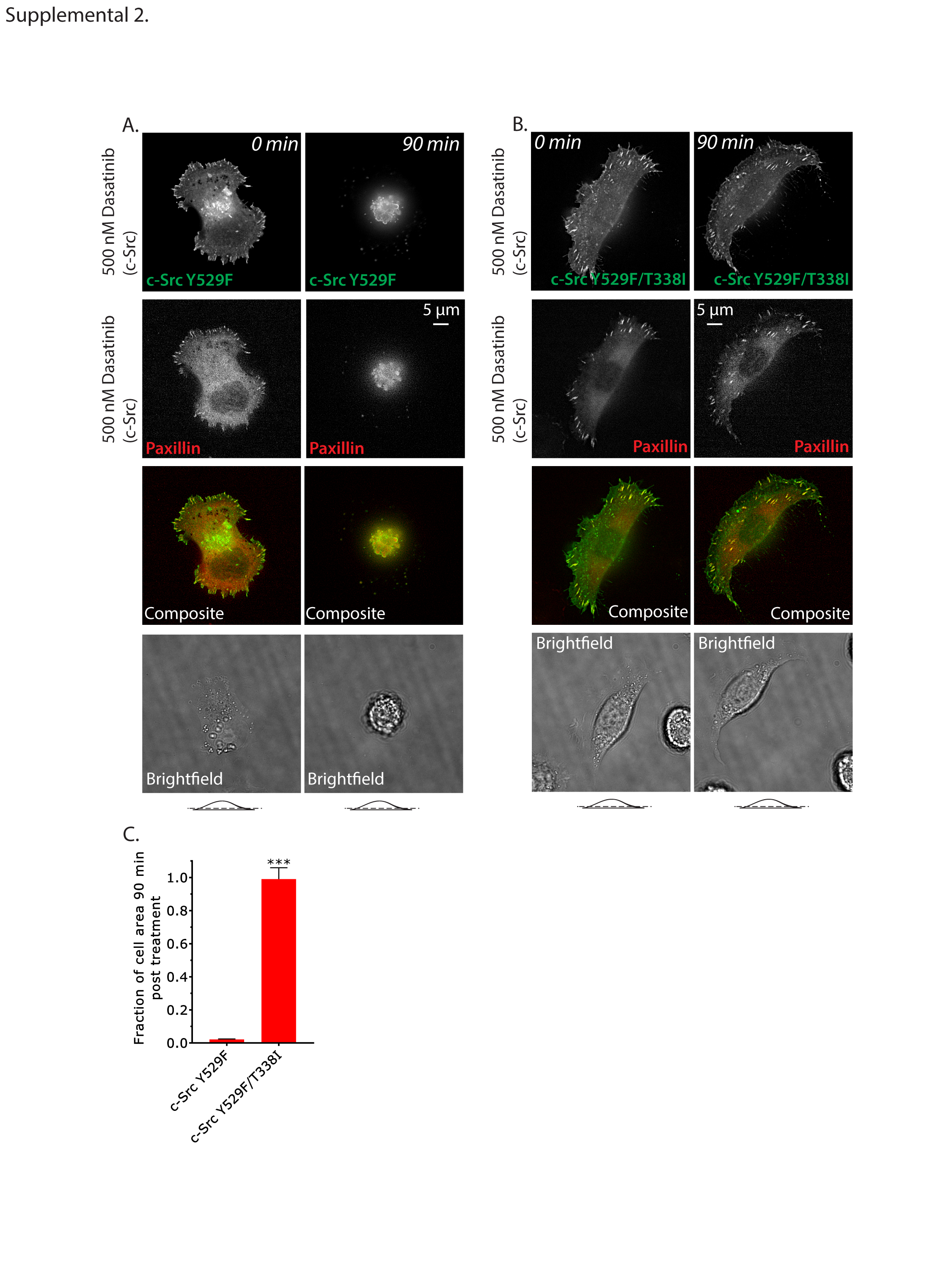

Supplement: Supplementary file 2 — S2 [file 41388_2017_71_MOESM2_ESM.tif]

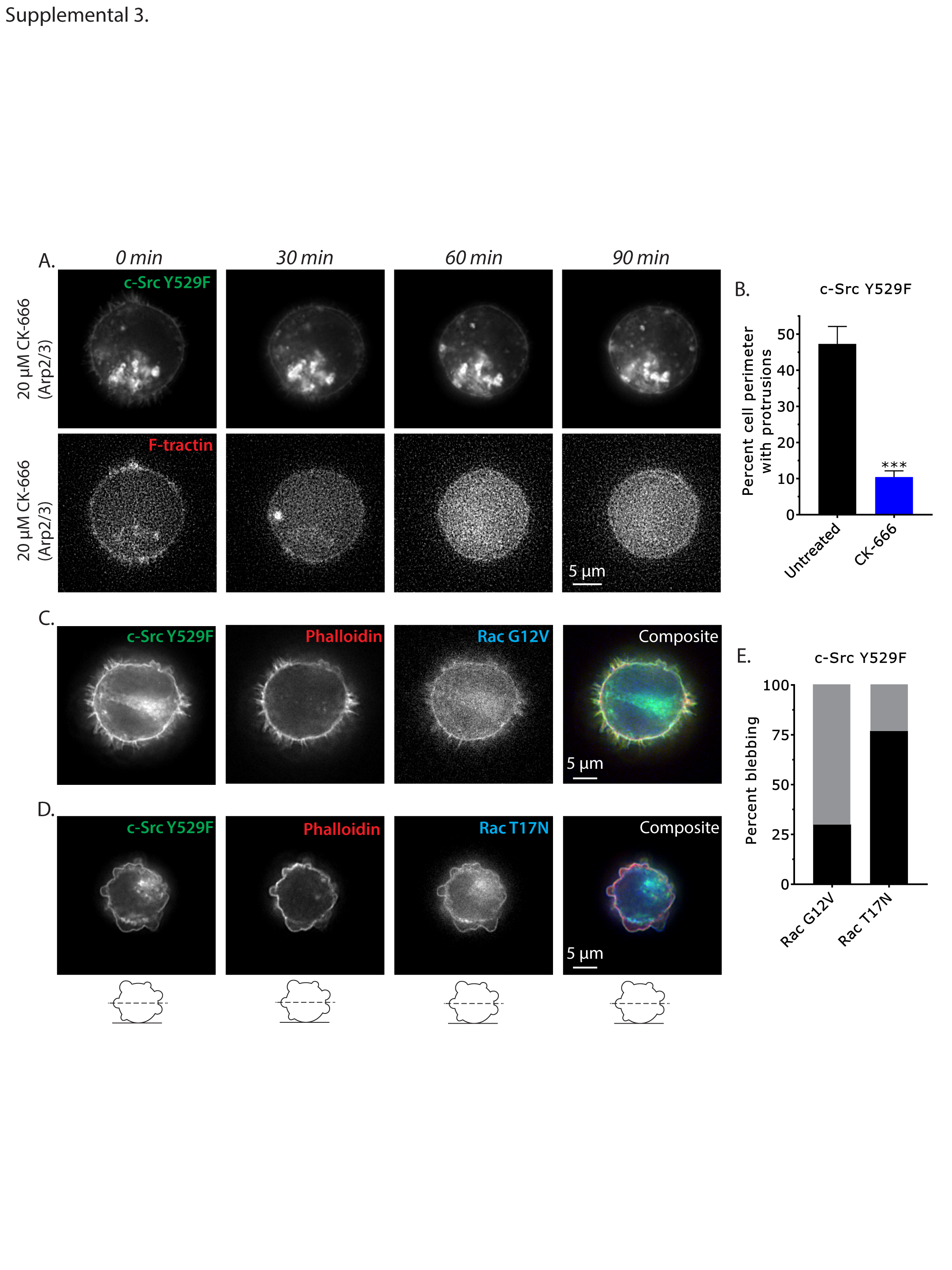

Supplement: Supplementary file 3 — S3 [file 41388_2017_71_MOESM3_ESM.tif]

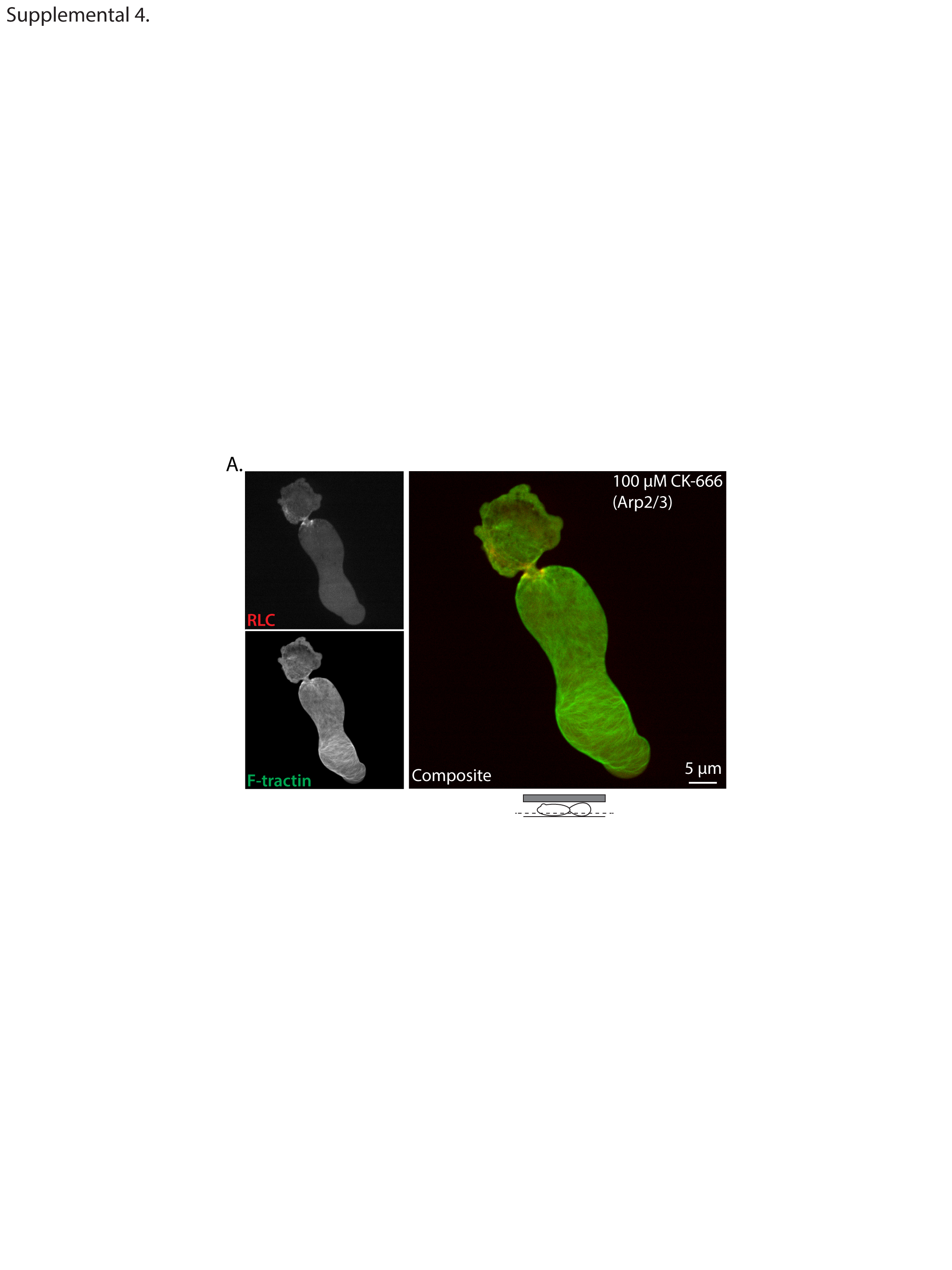

Supplement: Supplementary file 4 — S4 [file 41388_2017_71_MOESM4_ESM.tif]

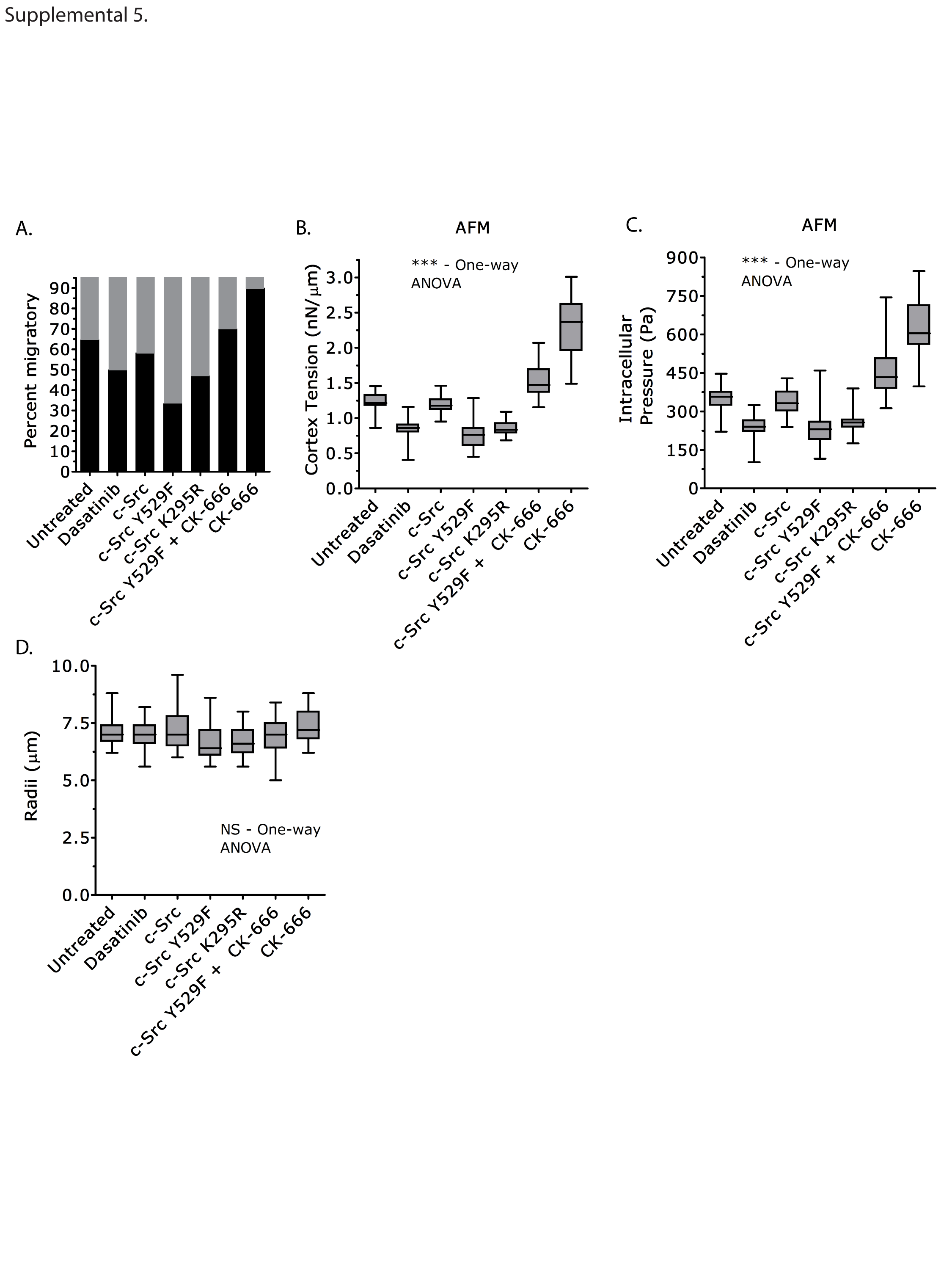

Supplement: Supplementary file 5 — S5 [file 41388_2017_71_MOESM5_ESM.tif]

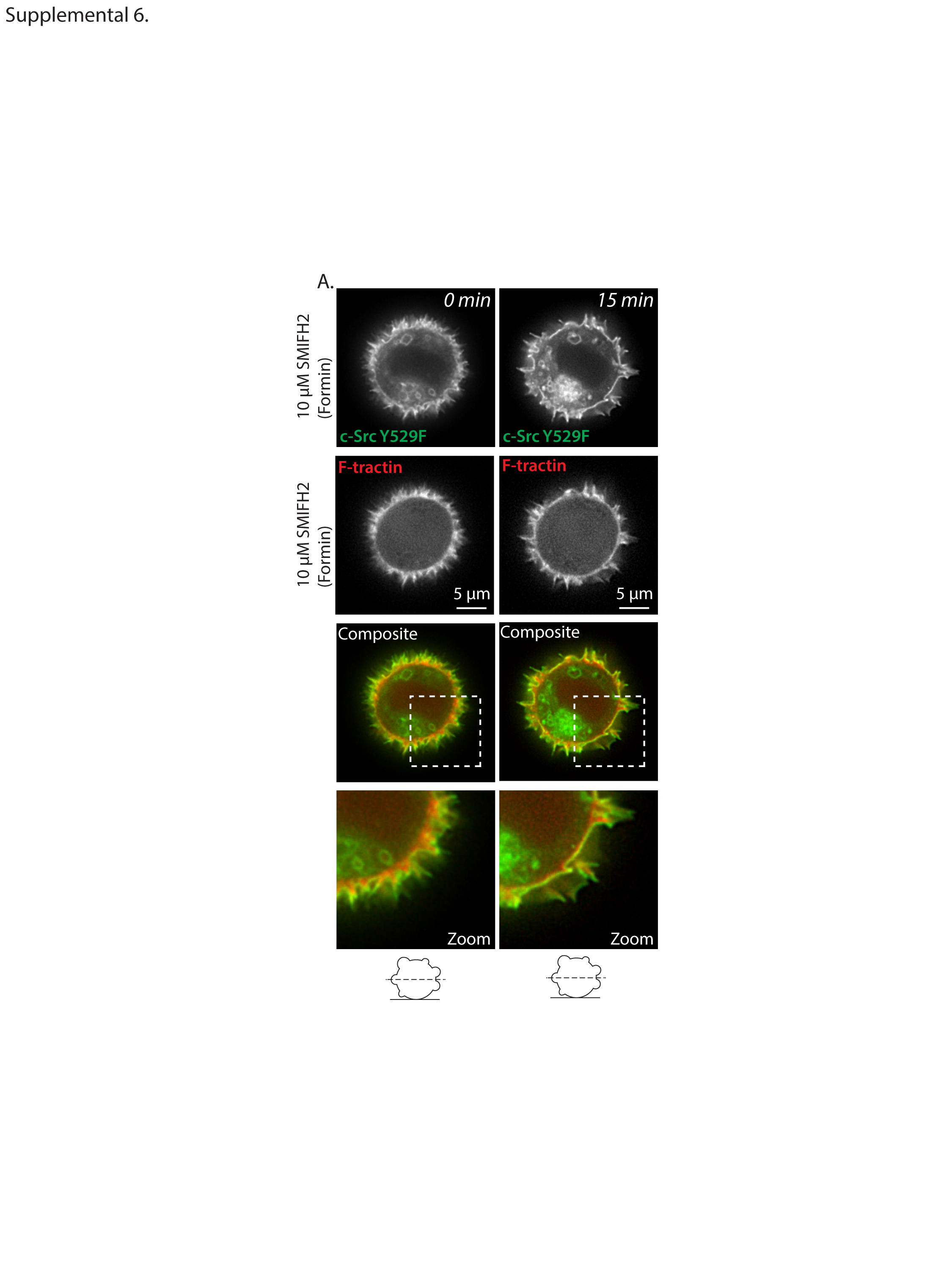

Supplement: Supplementary file 6 — S6 [file 41388_2017_71_MOESM6_ESM.tif]
