# Supplementary material for: c-Src activity is differentially required by cancer cell motility modes
Source: Oncogene. 2018 Jan 30;37(16):2104–21. doi: 10.1038/s41388-017-0071-5 (PMC5906457; doi:10.1038/s41388-017-0071-5)
Supplement: Supplementary file 20 — Supplementary Figure and Movie Legends [file 41388_2017_71_MOESM20_ESM.docx]

**SUPPLEMENTAL FIGURE LEGENDS**

**Supplemental figure 1. Targeted screen for pathway inhibitors that induces de-adhesion.** **A.** Time-lapse imaging of EGFP-paxillin expressing A375 cells on fibronectin coated (25 µg/mL) glass. All data are representative of at least two independent experiments. Drug was added at ‘0 min.’

**Supplemental figure 2. Dasatinib promotes de-adhesion of A375 cells by specifically inhibiting Src.** **A.** A375 cells adhered to fibronectin coated (25 µg/mL) glass expressing EGFP-c-Src^Y529F^ and Paxillin-FusionRed. Left column, before treatment (0 min) with Dasatinib. Right column, after treatment (90 min) with 500 nM Dasatinib. **B.** A375 cells adhered to fibronectin coated (25 µg/mL) glass expressing Dasatinib resistant EGFP-c-Src^Y529F/T338I^ and Paxillin-FusionRed. Left column, before treatment (0 min) with Dasatinib. Right column, after treatment (90 min) with 500 nM Dasatinib. **C.** Quantitative evaluation of ventral cell surface area after 500 nM Dasatinib treatment (10 cells per condition). Images were acquired using a GE DeltaVision Elite microscopy system. All data are representative of two independent experiments. Error is SEM. Statistical significance was determined by two-tailed Student’s t-tests. *** - p ≤ 0.001

**Supplemental figure 3. c-Src^Y529F^ promoted plasma membrane protrusions are inhibited by the Arp2/3 specific inhibitor CK-666 and dominant negative Rac^T17N^.** **A.** Time-lapse imaging of the central Z-section of A375 cells expressing EGFP-c-Src^Y529F^ and Paxillin-FusionRed on uncoated glass and treated with 20 µM CK-666. **B.** Quantitative evaluation of the percent cell perimeter with protrusions before (untreated) and 90 min after treatment with 20 µM CK-666 (10 cells). **C-D.** Central Z-section of EGFP-c-Src^Y529F^ expressing cells with constitutively active BFP-Rac^G12V^ (C) or dominant negative BFP-Rac^T17N^ (D) and stained for F-actin using fluorescently conjugated phalloidin. Images were acquired using a GE DeltaVision Elite microscopy system. All data are representative of two independent experiments. **E.** Quantitative evaluation of the percent blebbing cells expressing EGFP-c-Src^Y529F^ expressing cells with constitutively active BFP-Rac^G12V^ or dominant negative BFP-Rac^T17N^ (20 cells per condition). Error is SEM. Statistical significance was determined by two-tailed Student’s t-tests. *** - p ≤ 0.001

**Supplemental figure 4. Arp2/3 inhibition promotes the formation of large leader blebs. A.** Ventral Z-section of an EGFP-RLC and FusionRed-F-tractin expressing A375 cell highly confined under PDMS. CK-666 (100 µM) was added 30 min prior to highly confining cells. All data are representative of at least three independent experiments.

**Supplemental figure 5. Percent migratory, cortex tension, and intracellular pressure for all conditions. A.** Bar plot of the percent migratory cells (black bars; leader bleb-based) highly confined under PDMS (n=38, 15, 25, 32, 20, 18, and 22 cells, respectively). **B.** Box-and-whisker plot of cortex tension of freshly plated, round, A375 cells on uncoated glass for all conditions (n=28, 27, 21, 25, 27, 26, and 23 cells, respectively). **C.** Box-and-whisker plot of the intracellular pressure of freshly plated, round, A375 cells on uncoated glass for all conditions. **D.** Box-and-whisker plot of cell radii of freshly plated, round, A375 cells on uncoated glass for all conditions. All data are representative of at least three independent experiments. Statistical significance was determined by One-way ANOVA. *** - p ≤ 0.001

**Supplemental figure 6. Treatment of c-Src^Y529F^ expressing A375 cells with the formin inhibitor SMIFH2.** **A.** Central Z-section of A375 cells expressing EGFP-c-Src^Y529F^ and FusionRed-F-tractin on uncoated glass. Left column, before treatment (0 min) with SMIFH2. Right column, after treatment (15 min) with 10 µM SMIFH2. Images were acquired using a GE DeltaVision Elite microscopy system. All data are representative of two independent experiments. Zoomed images show protrusions taking on a wavy appearance after treatment with the formin inhibitor.

**Supplemental movie 1.** Control, untreated, widefield time-lapse imaging of an EGFP-paxillin

expressing A375 cell plated on fibronectin coated (25 µg/mL) glass.

**Supplemental movie 2.** Widefield time-lapse imaging of an EGFP-paxillin expressing A375

cell plated on fibronectin coated (25 µg/mL) glass. 500 nM Dasatinib was added at the time

indicated.

**Supplemental movie 3.** Spinning disk confocal time-lapse imaging of an EGFP-c-Src and

FusionRed-paxillin expressing A375 cell plated on fibronectin coated (25 µg/mL) glass.

**Supplemental movie 4.** Spinning disk confocal time-lapse imaging of an EGFP-c-Src^Y529F^ and

FusionRed-paxillin expressing A375 cell plated on fibronectin coated (25 µg/mL) glass.

**Supplemental movie 5.** Spinning disk confocal time-lapse imaging of an EGFP-c-Src and

FusionRed-paxillin expressing A375 cell plated on uncoated glass.

**Supplemental movie 6.** Spinning disk confocal time-lapse imaging of an EGFP-c-Src^Y529F^ and

FusionRed-F-tractin expressing A375 cell plated on uncoated glass.

**Supplemental movie 7.** Spinning disk confocal time-lapse imaging of an EGFP-c-Src^Y529F^ and

FusionRed-F-tractin expressing A375 cell plated on uncoated glass. 100 µM CK-666 was

added at the time indicated.

**Supplemental movie 8.** Ventral Z-section time-lapse imaging of an EGFP and

FusionRed-F-tractin expressing A375 cell highly confined under PDMS.

**Supplemental movie 9.** Central Z-section time-lapse imaging of an EGFP-c-Src and

FusionRed-F-tractin expressing A375 cell highly confined under PDMS.

**Supplemental movie 10.** Ventral Z-section time-lapse imaging of an EGFP-c-Src^Y529F^ and

FusionRed-F-tractin expressing A375 cell highly confined under PDMS.

**Supplemental movie 11.** Central Z-section time-lapse imaging of an EGFP-RLC and

FusionRed-F-tractin expressing A375 cell highly confined under PDMS.

**Supplemental movie 12.** Central Z-section time-lapse imaging of an EGFP-c-Src^Y529F^ and

FusionRed-RLC expressing A375 cell highly confined under PDMS.

**Supplemental movie 13.** Central Z-section time-lapse imaging of an EGFP-c-Src^K295R^ and

FusionRed-F-tractin expressing A375 cell highly confined under PDMS.
